# *PhiC31/PiggyBac* modified stromal stem cells: effect of interferon γ and/or tumor necrosis factor (TNF)-related apoptosis-inducing ligand (TRAIL) on murine melanoma

**DOI:** 10.1186/1476-4598-13-255

**Published:** 2014-11-26

**Authors:** Vahid Bahrambeigi, Nafiseh Ahmadi, Stefan Moisyadi, Johann Urschitz, Rasoul Salehi, Shaghayegh Haghjooy Javanmard

**Affiliations:** Applied Physiology Research Center, Isfahan University of Medical Sciences, Isfahan, Iran; Department of Genetics and Molecular Biology, School of Medicine, Isfahan University of Medical Sciences, Isfahan, Iran; Department of Anatomy, Biochemistry, and Physiology, John A Burns School of Medicine, University of Hawaii, Honolulu, HI 96819 USA; Manoa BioSciences, Honolulu, HI 96819 USA; Department of Physiology, Applied Physiology Research Center, School of Medicine, Isfahan University of Medical Sciences, Isfahan, Iran

**Keywords:** *PhiC31* integrase, *PiggyBac* transposase, Adipose derived mesenchymal stem cell, Interferon γ, TRAIL, Murine melanoma

## Abstract

**Background:**

TRAIL and IFNγ are promising anti-cancer cytokines and it has been shown that IFNγ may sensitize cancer cells to TRAIL. Adipose derived mesenchymal stem cells (ADSCs) are attractive vehicles for delivering anti-cancer agents. In this study, we evaluated the therapeutic potential of *PhiC31* (*φC31*) recombinase and/or *piggyBac* transposase (pB*t*) modified ADSCs expressing either TRAIL, IFNγ, or co-expressing TRAIL/IFNγ in mouse models of melanoma.

**Methods:**

The expression and bioactivity of mouse IFNγ and TRAIL in *φC31* and pB*t* modified cells were confirmed. We examined the effects of modified ADSCs on signal intensity of red fluorescence protein expressed by melanoma cells in subcutaneous tumors or established lung metastases and on survival (6 mice per group). We also conducted a flow cytometric analysis of systemic CD4^+^CD25^+^FOXP3^+^ T regulatory cells (Tregs) and histological analysis of melanoma tumors. Data were analyzed by Student t test, ANOVA, and log-rank tests. All statistical tests were two-sided.

**Results:**

We demonstrated non-viral DNA-integrating vectors can be used for stable transgene expression. IFNγ inhibited melanoma cell growth *in vitro* probably via IFNγ-induced JAK/STAT1 signaling pathway activation. Murine TRAIL induced apoptosis in the human cell lines CAOV-4 and Ej-138, while MCF7 and B16F10 cells appeared to be insensitive to TRAIL. Treatment of melanoma cells with IFNγ did not influence their response to TRAIL. In contrast, results from *in vivo* studies showed that IFNγ-expressing ADSCs, engrafted into tumor stroma, inhibited tumor growth and angiogenesis, prevented systemic increase of Tregs, increased PD-L1 expression and CD8+ infiltration (but not interleukin-2+ cells), and prolonged the survival of mice (68 days, 95% confidence interval [CI] =52 to 86 days compared to 36 days, 95% CI =29 to 39 days for control, *P* < .001).

**Conclusions:**

For the first time, we employed DNA integrating vectors for safe and stable modification of MSCs. Our data indicate potential of non-virally modified IFNγ-expressing ADSCs for treatment of melanoma through direct effects of IFNγ. This study may have a significant role in the management of cancer in the future.

**Electronic supplementary material:**

The online version of this article (doi:10.1186/1476-4598-13-255) contains supplementary material, which is available to authorized users.

## Background

Mesenchymal stem cells (MSCs) are emerging as promising tools for combined cancer gene/cell therapies since they have the unique ability of targeting tumor cells [1]. Several recent studies have successfully used viral-based gene transfer approaches to modify MSCs. However, immunogenicity, risk of insertional mutagenesis, and accidental production of self-replicating viruses are of concern and remain a problem for viral systems [2]. Non-viral gene delivery methods represent a simpler and safer alternative, as long-term expression of the therapeutic genes can be achieved though their stable integration into the host genome using DNA-based gene transfer vectors. Commonly used non-viral integrating vectors permanently integrate DNA into the host genome via either a recombinase or transposase [3]. *PhiC31 (φC31)* recombinase and *piggyBac* transposase (pB*t*) are two representatives of DNA-based gene transfer vectors that are under intensive development [4, 5]. The site-specific recombinase of bacteriophage *φC31* integrates the complete plasmid construct carrying an *attB* sequence into pseudo *attP* site in the mammalian genome [2]. Compared to *φC31*, pB*t* insert only the transposon cassette including the transgenes situated inside of terminal repeat elements (TREs) [6]. We used the *φC31* and pB*t* systems to achieve long term gene expression of therapeutic agents in murine adipose derived MSCs (ADSCs).

The cytokine type II interferon (IFNγ) can be used as a therapeutic agent as it exerts a variety of different anti-tumor effects, including inhibition of cancer cell proliferation, repression of tumor angiogenesis, and the induction of tumor cell apoptosis [7, 8]. IFNγ also stimulates the host immune response and enhances tumor cell apoptosis via tumor necrosis factor (TNF)-related apoptosis-inducing ligand (TRAIL) [9]. TRAIL in its role as a death ligand binds to the surface death receptors (DR; DR1 and DR2) and induces apoptosis in a variety of neoplastic cells while sparing most normal cells. Cancer cells have variable levels of sensitivity to TRAIL-mediated apoptosis [10] and studies have shown that IFNγ pre-treatment can sensitize some of the resistant cancer lines to TRAIL [11–15]. Besides, IFNγ/TRAIL combination immunotherapy has been shown to synergistically induce tumor cell death [16]. However, to yield significant anti-tumor activity, multiple high-dose systemic administration of these cytokines is necessary which is associated with adverse side effects [10, 17]. To overcome this limitation, several studies used cytokine-expressing MSCs to mitigate cancer progress in tumor models including melanoma [18–20]. Therefore, in this study we aimed to investigate antitumor activity of *φC31*/pB*t* modified murine ADSCs expressing IFNγ and TRAIL individually, or co-expressing Trail/IFNγ *in vitro* and in mouse subcutaneous or lung metastasis models of melanoma.

## Results

### Characterization of murine ADSCs

**Figure 1:**
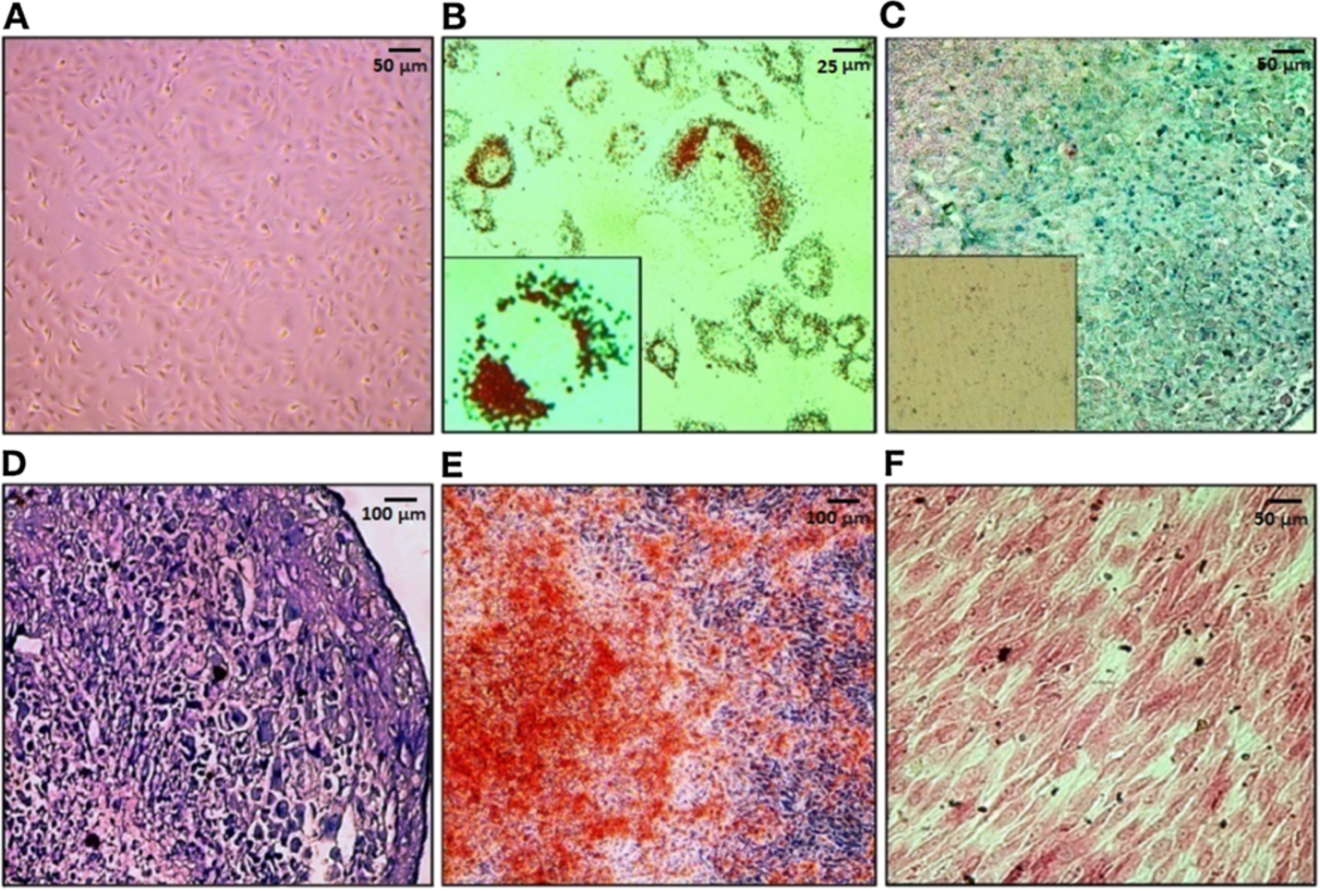
**Differentiation potential of murine adipose derived mesenchymal stem cells (ADSCs). A)** Isolated ADSCs at passage 6. **B)** Differentiation of murine ADSCs into adipocytes. Oil red O staining visualizes lipid vacuoles. **C** and **D)** Cartilage differentiation of ADSCs. Alcian blue **(C)** and Toluidin blue staining **(D)** indicate successful differentiation of ADSCs into chondrocytes. Inlay shows negative control for Alician blue staining. **E** and **F)** Osteogenic differentiation was confirmed by Alizarin red S **(E)** and Von Kossa staining **(F)**. Red coloring in Alizarin red S and black nodules in Von Kossa staining respectively, indicative of calcium and extra matrix deposits produced by ADSCs. ADSC = adipose derived mesenchymal stem cell.

**Figure 2:**
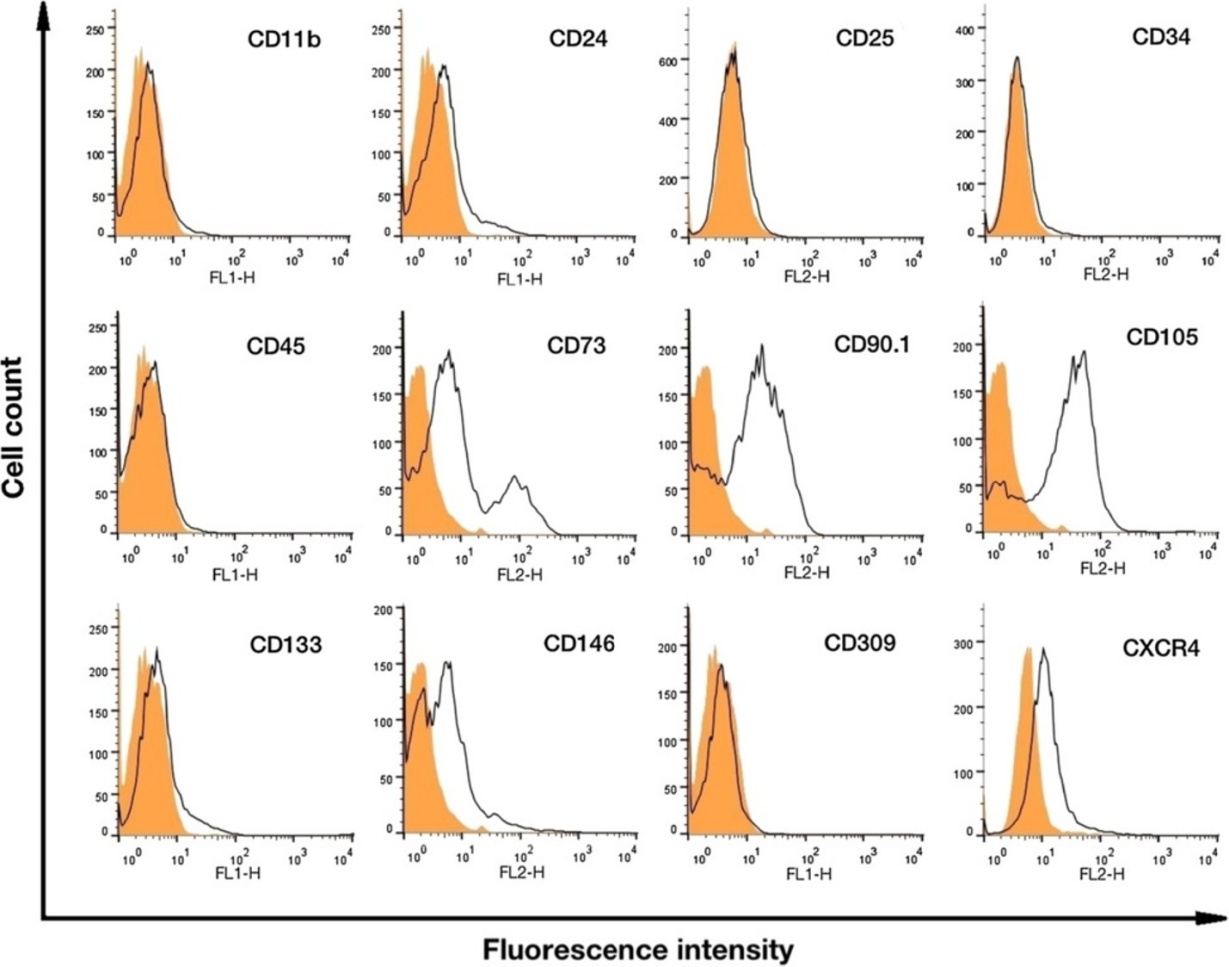
**Representative fluorescence-activated cell sorting (FACS) analysis of surface markers expressed by murine ADSCs cultured at Passage 6.** One experimental representative of three is shown. Cells were incubated with different monoclonal antibodies conjugated with either fluorescein isothiocyanate (FITC) or phycoerythrin (PE). White histograms correspond to cells stained with antibodies against mouse surface epitopes. Staining with the appropriate IgG isotype control antibody is shown in orange. Murine ADSCs were positive for stem cell markers like CD73, CD90.1, CD105, CD146 and MSC-homing marker CXCR4. A small sub-population of ADSCs also expressed stem cell markers like CD24 and CD133. ADSCs were negative for hematopoietic markers like CD11b, CD25, CD34, CD45 and the endothelial marker CD309. ADSC = adipose derived mesenchymal stem cell; IgG = immunoglobulin G; CXCR4 = Chemokine Receptor type 4.

The authenticity of ADSCs was verified by differentiation experiments (Figure 1) along with immunophenotypic analysis of surface antigenes (Figure 2). ADSCs were isolated based on their adherence to the surface of culture dishes. Isolated cells expanded rapidly and in the third passage uniformed cells were obtained. Cells from passage 6 were used for characterization experiments. Plasticity of ADSCs was confirmed by differentiation of isolated ADSCs (Figure 1A) to adipocytes (Figure 1B), chondrocytes (Figure 1, C and D) and osteoblasts (Figure 1, E and F). Appearance of red colored lipid vacuoles in Oil red O staining, green colored mucopolisaccarides in Alcian blue staining, purple colored proteoglycans in Toluidin blue staining, red colored calcium deposits in Alizarin red staining and black colored mineralized deposits in Von Kossa staining demonstrated successful differentiation of ADSCs into three different cell lineages. Immunotyping of ADSCs revealed expression of stem cell markers CD73, CD90.1, CD105, CD146 and MSC-homing marker CXCR4. Moreover, a small sub-population of ADSCs showed a weak expression of stem cell markers CD24 and CD133. We did not detect any expression of surface markers CD11b, CD25, CD34, CD45 and CD309, confirming that our ADSC preparations did not contain any hematopoietic or endothelial cells (Figure 2).

### Stable modification of ADSCs and melanoma B16f10 Cells by DNA integrating vectors

**Figure 3:**
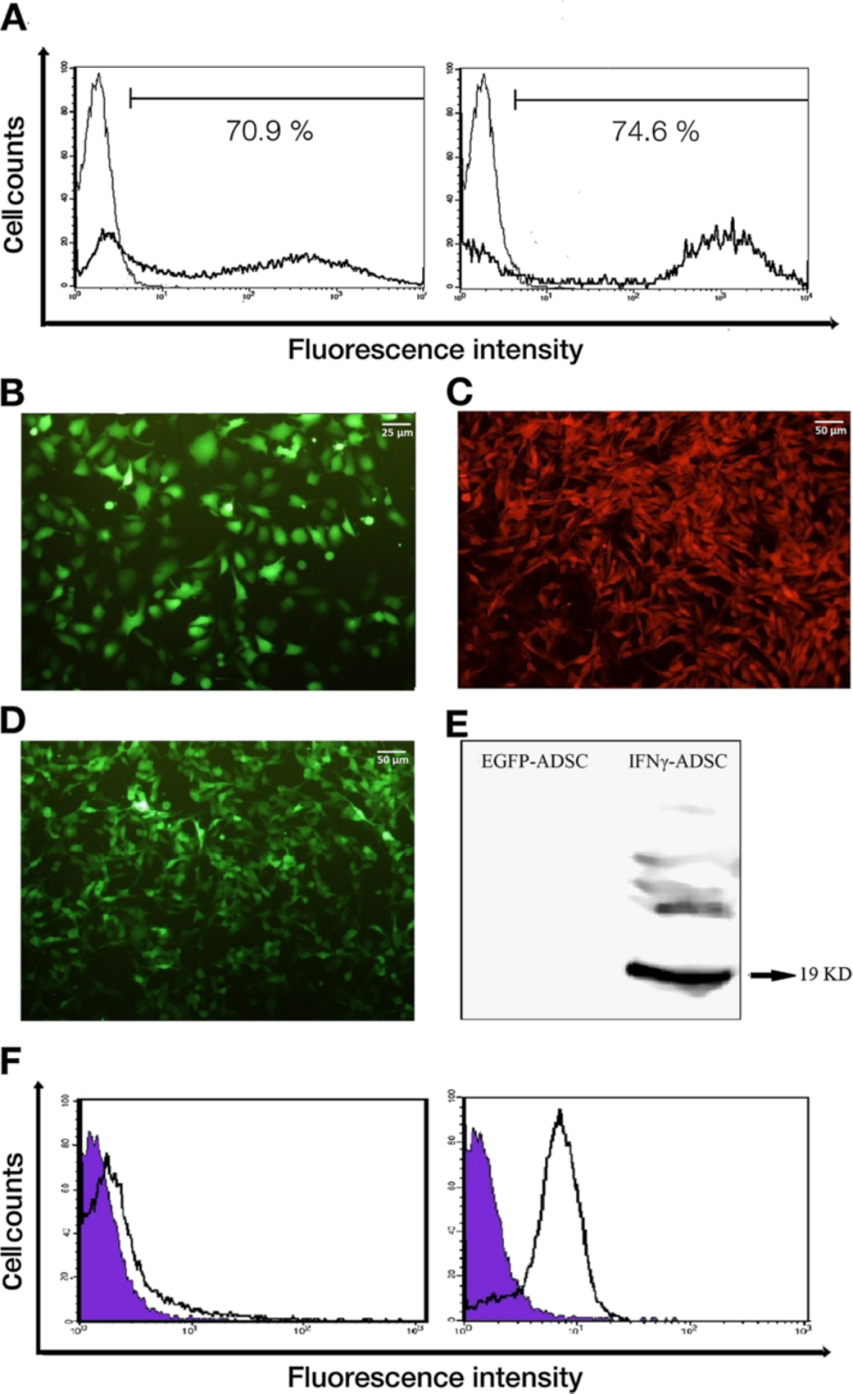
**Stable modification of ADSCs and melanoma B16f10 cells with the use of DNA integrating vectors. A)** Flow cytometric analysis of EGFP expression by ADSCs. (Left plot) Mean percentage of EGFP-expressing ADSCs post 48 h of nucleofection with pmaxGFP vector, indicating efficient transgene transfer into mouse ADSCs. (Right plot) Mean percentage of EGFP-positive ADSCs two weeks after nucleofection with p*mhy*GENIE-3 and hygromycin selection. Results are representatives of triplicate experiments. **B)** Fluorescence microscopy image of EGFP-expressing ADSCs after nucleofection with p*mhy*GENIE-3 and two weeks selection with hygromycin. **C)** Stable RFP-expressing melanoma cells after lipofection with pDsRed-attb-zeo/pCMVInt and three weeks of zeocin selection. **D)** EGFP-expressing melanoma cells after lipofection with pBEB/pCMVInt and three weeks of geneticin selection. **E)** Western blot analysis of IFNγ from ADSC cell lysates. Modified ADSCs were selected with hygromycin following nucleofection with either p*mhy*GENIE-3 (Lane 1) or p*mhy*GENIE-3-IFNγ (Lane 2). Blotting was performed using a monoclonal antibody against mouse IFNγ. **F**) Cytofluorimetric evaluation of TRAIL expression in ADSCs selected with geneticin following co-nucleofection with pBEB/pCMVInt (Violet histograms) and pBTB/pCMVInt (white histograms). ADSCs were stained with a PE conjugated monoclonal antibody to mouse TRAIL. A single representative experiment of triplicates is shown. (Left plot) Surface staining of TRAIL-ADSCs after 48 h of co-nucleofection. (Right plot) Surface staining of TRAIL-ADSCs after co-nucleofection and three weeks of geneticin selection. PE = phycoerythrin.

There is little information available regarding nucleofection of murine ADSCs. In this study, we achieved a transfection efficiency of ~71% using the Human MSC Nucleofector kit with program X-001 and plasmid pMax (a vector expressing EGFP) (Figure 3, A, Left). ADSCs expressing enhanced green fluorescent protein (EGFP), IFNγ, TRAIL and co-expressing IFNγ/TRAIL are respectively referred to EGFP-ADSC, IFNγ-ADSC, TRAIL-ADSC and IFNγ/TRAIL-ADSC. FACS analysis of ADSCs nucleofected with p*mhy*GENIE-3-EGFP revealed that ~75% of ADSCs were EGFP-positive after two weeks of culture under antibiotic selection (Figure 3, A, Right). Furthermore, a microscopic evaluation of these cells confirmed their efficient modification (Figure 3, B). Melanoma B16F10 cells, stably transformed and grown for 2 weeks under geneticin or zeocin selection, uniformly expressed Red fluorescent protein (RFP) (Figure 3, C) and EGFP (Figure 3, D) respectively (referred as RFP-melanoma and GFP-melanoma). High levels of IFNγ expression were confirmed by Western blot analysis as indicated by a 19 kD band in IFNγ-ADSCs (Figure 3, E). FACS analysis of cells stained with fluorescently-labeled anti-TRAIL antibody confirmed a relevant protein expression in TRAIL-ADSCs and the absence of TRAIL in EGFP-ADSCs (Figure 3, F).

### Effect of IFNγ on viability and apoptosis of melanoma cells and the possible involvement of JAK/STAT1 dependent signaling pathway

**Figure 4:**
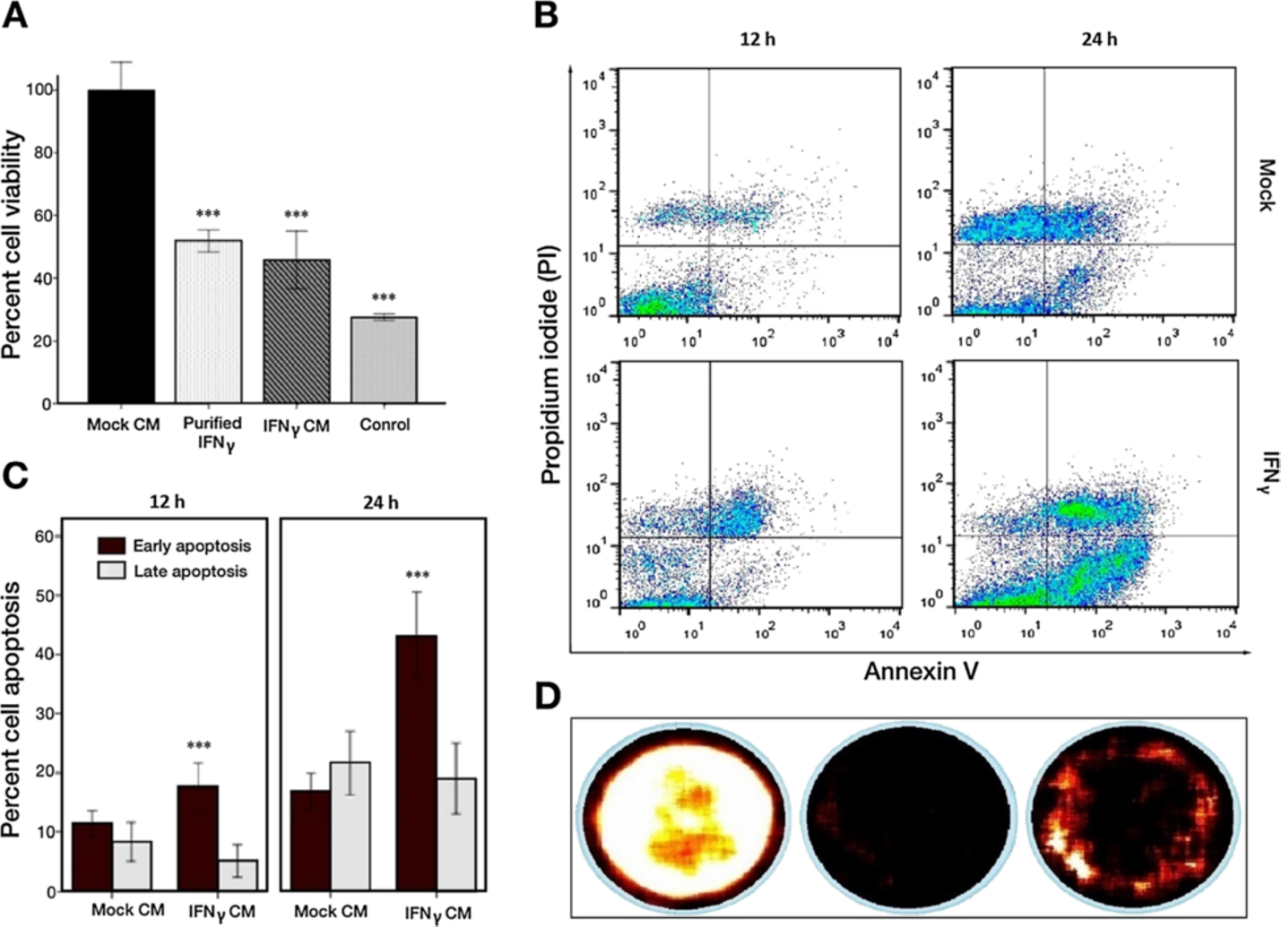
**The bioactivity of IFNγ secreted from IFNγ-ADSCs. A)** The sensitivity of melanoma B16F10 cells to IFNγ was evaluated by the MTT cell viability assay. Melanoma cells were incubated in CM (culture medium) obtained from either EGFP-ADSCs (mock) or IFNγ-ADSCs for 3 days. Zeocin (10^4^ μg/mL) and purified mouse IFNγ (1000 pg/mL) were used respectively as a positive and sample control (all samples are compared to mock). Cell viability after treatment is presented as a percentage relative to mock treated cells. ***P < .001. **B)** Representative FACS plots from Annexin V/propidium iodide (PI) apoptosis assay showing an increase in early apoptosis of melanoma cells when cells are incubated in CM from IFNγ-ADSCs (Lower panels) compared to CM from EGFP-ADSCs (Upper panels) for 12h and 24h incubation. One experimental representative of triplicates is shown. **C)** A bar graphs summarizing the FACS results from annexin V/PI apoptosis assay in two time points demonstrating statistically significant increase in early apoptosis of melanoma cells in the presence of IFNγ. Data represent mean values of three replicates in three independent experiments. ***P < .001. **D)** Monitoring the induction of the STAT1 and STAT2 components of Jak/STAT-mediated signal transduction pathways. Melanoma cells were stably modified to express Firefly luciferase (FL) under CMV enhancer-CAG promoter (FL-melanoma), or under direct repeats of the interferon-stimulated response element (ISRE) and a minimal TA promoter (ISRE/FL-melanoma). ISRE/FL-melanoma cells were incubated in CM obtained from either EGFP-ADSCs (mock) or IFNγ-ADSCs for 48 hours and then ONE-Glo™ luciferase assay reagent was added to each sample. Luminescence imaging showed IFNγ-induced Jak/STAT-mediated signal activation in melanoma B16F10 cells. (Left) FL-melanoma cells which were used as a positive control. (Middle) ISRE/FL-melanoma cells incubated with supernatants from EGFP-ADSCs. (Right) ISRE/FL-melanoma cells incubated with CM from IFNγ-ADSCs. One experimental representative of triplicates is shown.

To evaluate the effect of IFNγ on the proliferation of melanoma cells we incubated B16F10 cells with 100 μl of culture medium (CM) supernatant from IFNγ-ADSCs. We determined the IFNγ concentration by ELISA at about 1800 pg per mL of CM. We evaluated the Melanoma cell proliferation and apoptosis by the 3-(4,5-Dimethylthiazol-2-yl)-2,5-diphenyltetrazolium bromide (MTT) and Annexin V apoptosis assays. For MTT assay, melanoma cells were incubated in CM from either EGFP-ADSCs or IFNγ-ADSCs. Zeocin, a proliferation inhibitor, was used as a positive control (10^4^ μg/mL) to monitor apoptosis. Recombinant mouse IFNγ was used to evaluate the effect of purified IFNγ on the proliferation of melanoma cells. All samples were compared to mock. The results from MTT assays, shown in Figure 4A demonstrated a statistically significant decrease in viability of these melanoma cells, similar to the bioactivity effect of purified mouse IFNγ (ANOVA, *P* < .001; Mean percent are shown on Additional file 1: Table S1). As a subsequent step, we assessed the potential of IFNγ on apoptosis of melanoma cells using Annexin V apoptosis assay. Results shown in Figure 4, B and C indicated that incubation of melanoma cells with IFNγ-containing CM induces early apoptosis of melanoma cell over the incubation period in a statistically significant manner (ANOVA, *P* < .001). The mean percentage of early apoptosis in melanoma cells during the incubation period of 24 hours with supernatant from IFNγ-ADSC increased from ~18 to ~43% while melanoma cells treated with mock supernatant showed only ~11 to ~17% of early apoptosis (Additional file 1: Table S2). Additionally, we investigated whether IFNγ produced by IFNγ-ADSCs can activate the JAK/STAT1 dependent signaling pathway in melanoma cells. For this purpose we prepared two types of permanently modified melanoma B16F10 cells. The first set of cells contained the firefly luciferase gene under direct repeats of the interferon-stimulated response element (ISRE) and a minimal TA promoter (referred herein as ISRE/FL-melanoma cells). The second set of melanoma cells were modified to constitutively express firefly luciferase under the control of a cytomegalovirus (CMV) enhancer, a chicken beta actin and rabbit beta globin intron (CAG) promoter (referred to as FL-melanoma cells and used as positive control cells). FL-melanoma and ISRE/FL-melanoma cells were incubated with supernatants from either EGFP-ADSCs or IFNγ-ADSCs for 48 hours. The analysis of the visible luminescence signal intensity clearly demonstrated IFNγ-induced Jak/STAT-mediated signal activation in ISRE/FL-melanoma cells (Figure 4D).

### Bioactivity of TRAIL and *In Vitro*effect of IFNγ on sensitization of melanoma cells

**Figure 5:**
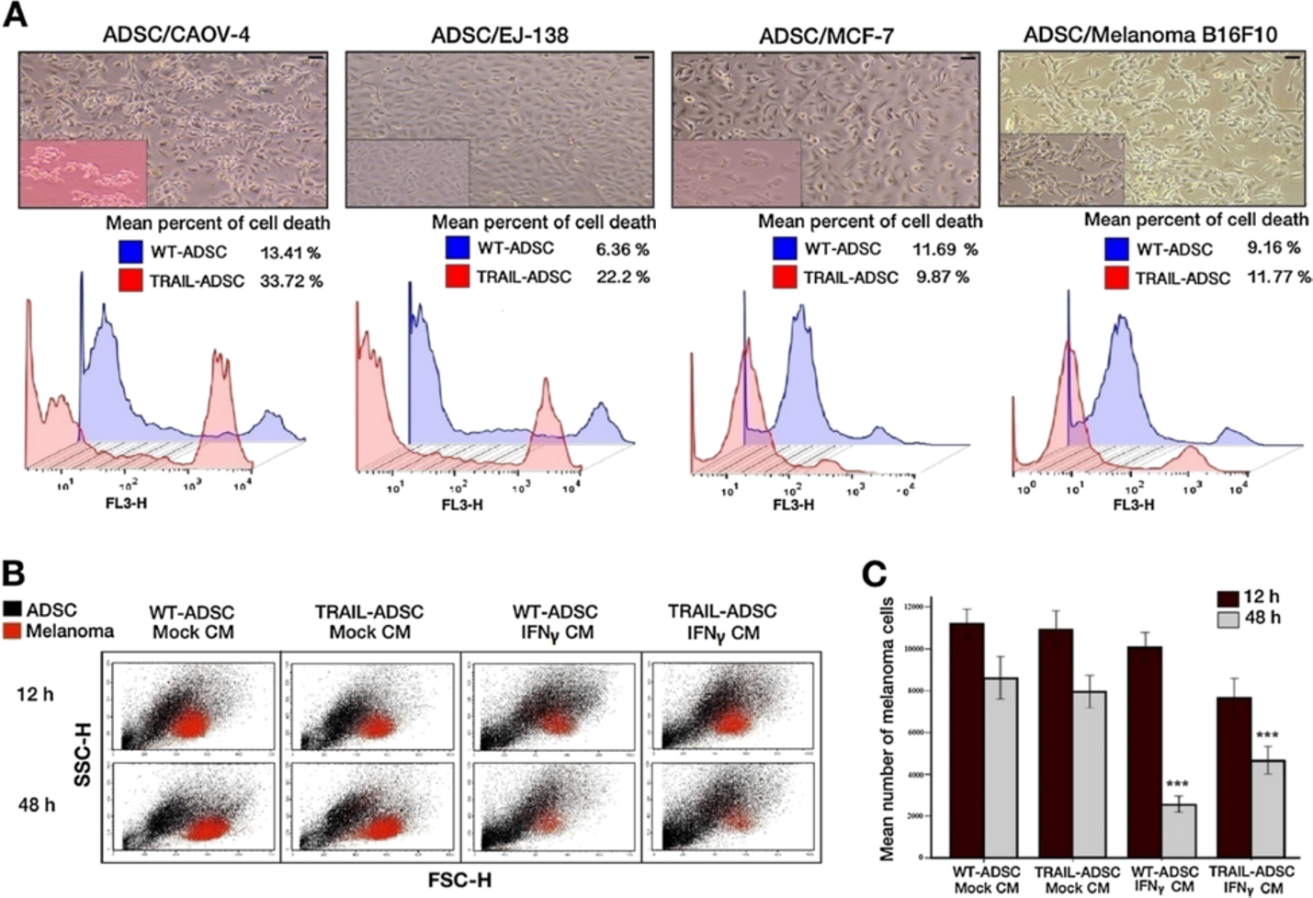
**Apoptotic potential of TRAIL-producing ADSCs and the effect of IFNγ on sensitivity of melanoma B16F10 cells to TRAIL in co-culture experiments. A)** WT-ADSCs and TRAIL-ADSCs co-cultured with CAOV-4, Ej-138, MCF-7 and B16F10. Upper panel: Representative microscopy images from each one of triplicate co-culture experiments. Inlays show the morphology of each cancer cell line. Lower panel: Representative FACS plots showing a statistically significant increase in dead CAOV-4 and Ej-138 cells (Student t test*, P* < .001), implying bioactivity of TRAIL produced by murine modified ADSCs. Each plot represents a mean value of three independent experiments. *P* values are two-sided. Scale bars =50 μm. **B)** FACS plots from co-culture experiments demonstrated a decrease in proliferation of red fluorescent protein (RFP)-expressing melanoma cells in groups co-cultured with WT-ADSCs and TRAIL-ADSCs only in the presence of IFNγ. Population of ADSCs and melanoma cells are shown in black and red respectively. One experimental representative of triplicate independent experiments is shown. **C)** A FACS analysis (results depicted as bar graph) demonstrated a statistically significant decrease in melanoma cell number co-cultured with both WT-ADSCs and TRAIL-ADSCs after addition of IFNγ-containing CM. However, the effect of IFNγ at 48 h of incubation on melanoma cells co-cultured with WT-ADSCs was more significant (compared to melanoma cells co-cultured with TRAIL-ADSCs), implying IFNγ does not effect the activity of TRAIL on melanoma B16F10 cells. Experiment was performed in triplicate**.** Error bars correspond to 95% confidence intervals. *P* values are two-sided. ***P < .001 (ANOVA). WT = wild type.

To explore the bioactivity of TRAIL, triplicate co-culture experiments were performed. Wild type ADSCs (WT-ADSCs) or TRAIL-ADSCs were co-cultured with CAOV-4, Ej-138, MCF-7, or B16F10 cells (Figure 5A). We observed an effect of murine TRAIL in CAOV-4 and Ej-138 lines. The mean mortality rate increased in a statistically significant manner from 13.41 ± 1.2 % to 33.72 ± 5.1 % and 6.36 ± 0.6 % to 22.2 ± 2.4 % for CAOV-4 and Ej-138 respectively (t-test, *P* < .001). TRAIL-ADSCs had no effect on the other cell lines (t-test, *P* = .545 for MCF-7 and *P* = .28 for B16F10). For the next step, we co-cultured melanoma cells with either WT-ADSC or TRAIL-ADSC, in mock CM or IFNγ-containing CM, and for 12 h and 48 h (Figure 5B). The results demonstrated that a 48 h incubation of melanoma cells with either WT-ADSC or TRAIL-ADSC in the presence of IFNγ reduced the melanoma cell numbers (ANOVA, *P* < .001). Shorter incubation periods with or without IFNγ had no effect on melanoma cell numbers (Figure 5C).

### Plasma levels of IFNγ after injection of IFNγ-producing ADSCs to mice

ELISA was performed to evaluate the effect of stem cell therapy on systemic levels of IFNγ at three time points (days 10, 16, and 21). The systemic levels of IFNγ in all of the experimental groups were below the detection level of our ELISA assay, indicating very low plasma levels.

### *In Vivo*effect of modified ADSCs on growth of subcutaneous melanoma and melanoma pulmonary tumors, and survival analysis of mice with melanoma metastases

**Figure 6:**
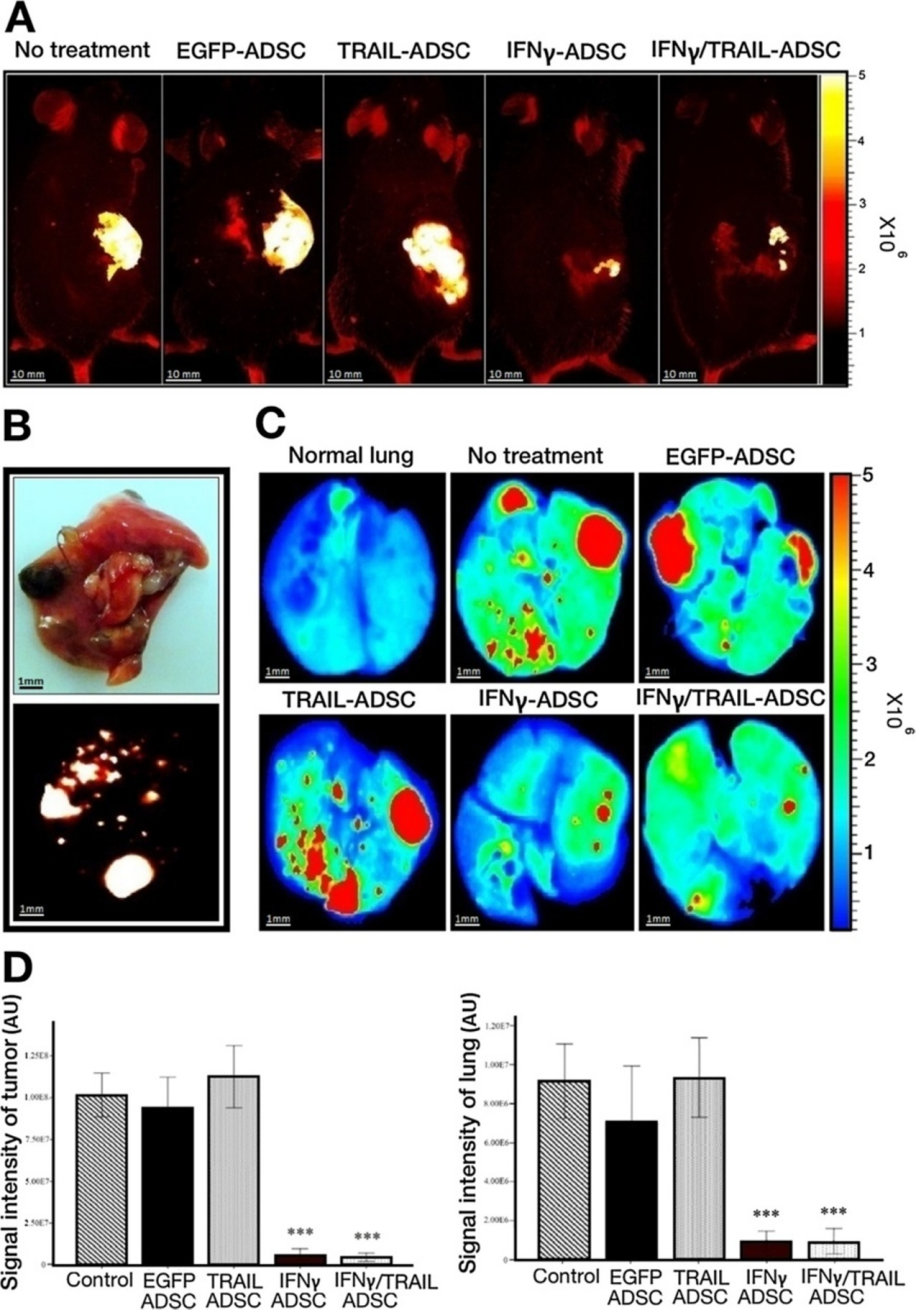
***In vivo*** **and** ***ex vivo*** **red fluorescence optical imaging obtained with the In-Vivo FX Pro small-animal imaging system. A)** Representative red fluorescence *in vivo* images of subcutaneous melanoma bearing mice in different treatment groups. Red fluorescence protein (RFP)-expressing melanoma cells in density of 2 × 10^6^ were delivered either alone or concurrently with 7.5 × 10^5^ of genetically modified ADSCs (GM-ADSCs). Mice were anesthetized with isoflurane and images were captured using a multi-wavelength source with a 2 min exposure time. **B)** Upper image showing a lung with RFP-melanoma lung metastases at day 28. Lower image, red fluorescence optical imaging of the same lung, indicating that some of RFP-melanoma metastases are not superficially visible. **C)** Representative red fluorescence *ex vivo* images of dissected lungs in different treatment groups. On day 0, 7.5 × 10^5^ of RFP-melanoma cells were injected into the lateral tail vein of mice. Ten days later, mice were given PBS or 7.5 × 10^5^ of GM-ADSCs by tail vein injection. Mice were sacrified at day 28 for *ex vivo* optical imaging of lung samples, performed using a multi-wavelength source with a 2 min exposure time. **D)** Quantification of fluorescent signal intensities was performed using the Bruker Molecular Imaging Software. Fluorescence intensity was expressed as arbitrary units (AU) and was reported as a mean ± SD in the bar graphs. (Left) Quantification of *in vivo* images (photons/s of subcutaneous tumor region). Bar graphs indicate significant subcutaneous tumor regression in mice that were treated with IFNγ-expressing ADSCs, whereas tumors grew rapidly in the other groups. (Right) Quantification of *ex vivo* images (photons/s of lung region). Bar graphs demonstrating reduced red fluorescence intensity in lung samples from mice injected with IFNγ-expressing ADSCs, while red fluorescence intensity significantly increased in other groups. Each group consisted of six mice. ***P < .001 (ANOVA).

**Figure 7:**
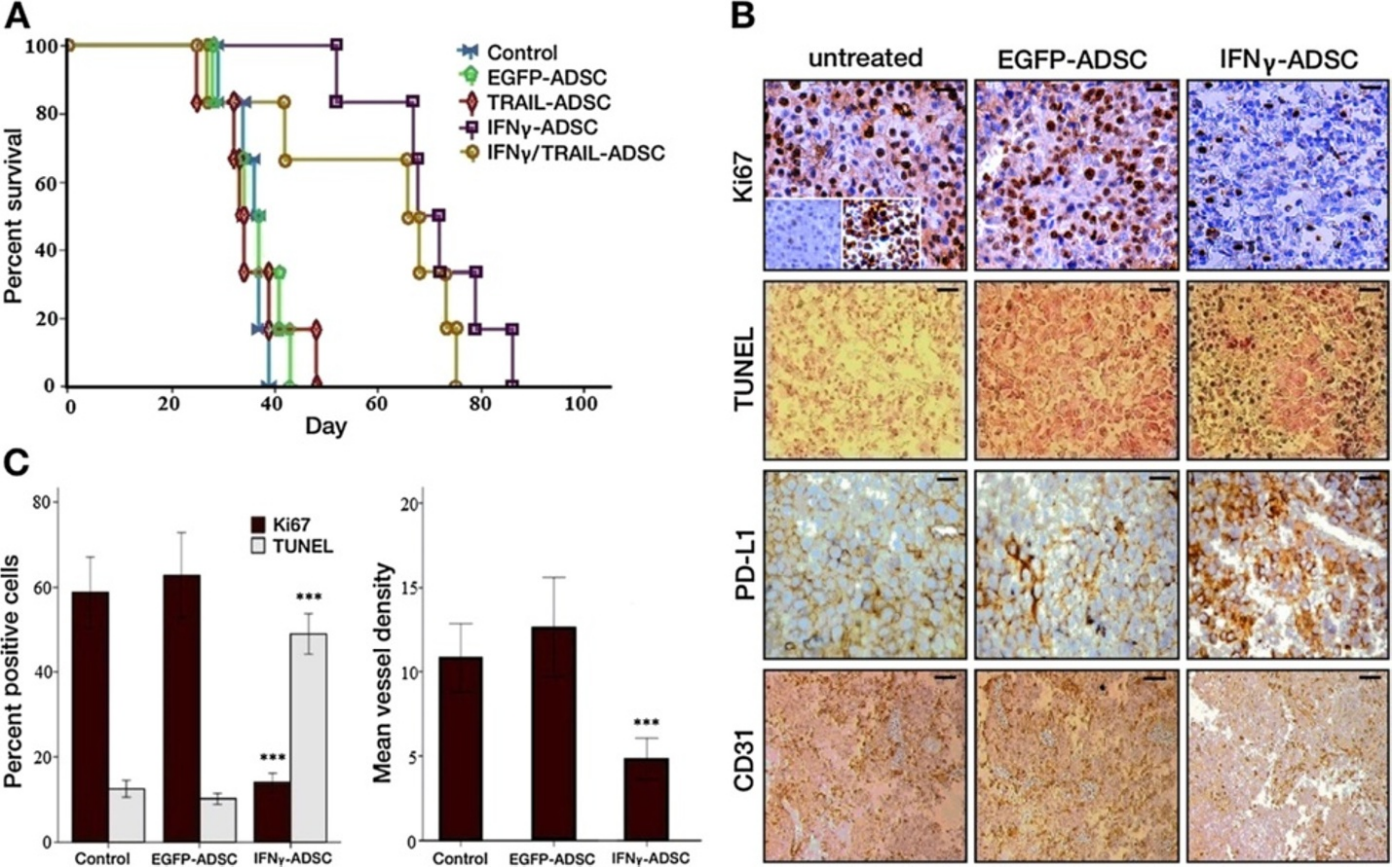
**Long-term survival and melanoma lung tumor analysis. A)** Survival of mice with established pulmonary metastases of B16F10 melanoma cells, intravenously injected with genetically modified ADSCs. An additional group of mice with established lung metastases derived from melanoma cells received only PBS (control group). Survival was measured from the day of melanoma cell injection until the day of death. Survival curves were drawn by the Kaplan-Meier method (n =6 in each treatment group). **B)** Representative microscopic images of immunohistochemistry (IHC) staining for cell proliferation marker Ki67 (Inlays show negative control and positive controls), TUNEL staining for analysis of tumor cell apoptosis, IHC staining for PD-L1 (Scale bars =50 μm), and IHC satining for neovasculature marker CD31 (Scale bar =100 μm). Microscopic images clearly shows injected IFNγ-expressing ADSCs significantly upregulate PD-L1 expression in melanoma lung tumor cells (compared to untreated control and EGFP-ADSC injected groups). **C**) Bar graphs showing statistically significant effect of IFNγ-ADSCs on growth of metastatic melanoma tumors. (Left) Quantification of proliferation and apoptosis was performed by averaging percentage of positively stained cells to total cells within 10 randomly selected areas at x200 magnification. IFNγ-producing ADSCs significantly decreased the melanoma cell proliferation and increased tumor cell apoptosis in groups treated with IFNγ-producing ADSCs in a statistically meaningful manner (ANOVA; *p* < .001). (Right) Quantification of angiogenesis was performed by counting the number of vessels in 10 randomly selected areas of CD31 stained sections at x200 magnification. Results indicate statistically significant inhibition of angiogenesis after injection of IFNγ-ADSCs. Each group consisted of four mice. ***P < .001 (ANOVA). The statistical tests were two-sided. PDL1 = programmed cell death 1 ligand 1.

We examined the effects of modified ADSCs on signal intensity of red fluorescence protein expressed by melanoma cells in subcutaneous tumors or established lung metastases and on survival. For each model, mice were randomly divided into the following five experimental groups: injected with (a) PBS (control group), (b) EGFP-ADSCs, (c) TRAIL-ADSCs, (d) IFNγ-ADSCs and (e) IFNγ/TRAIL-ADSCs. Each group consisted of 6 mice and the experiment for metastasis models was performed in duplicate. There was no observed adverse health effect related to the injection of ADSCs. *In vivo* optical imaging of mice with subcutaneous melanoma revealed a statistically significant reduction of melanoma tumor growth detected by red fluorescent signal in groups co-injected with IFNγ-expressing ADSCs (ANOVA, *P* < .001, Figure 6A). There was no additive effect on tumor ablation when IFNγ/TRAIL-ADSCs were co-injected with the melanoma cells, indicating that presence of IFNγ is solely responsible for tumor reduction (Figure 6, A and D, left panel). Co-inoculation of melanoma cells with EGFP-ADSC (ANOVA, *P* = .592) or TRAIL-ADSC (*P* = .798) did not alter tumor growth. In lung metastatic models, metastatic colonies were observed in all treatment groups. Using *ex vivo* imaging we noticed the presence of several internal colonies that were not visible on the surface of the lungs (Figure 6B). However, the colony size and number was reduced in mice treated with IFNγ-ADSCs and IFNγ/TRAIL-ADSCs in a statistically significant manner (ANOVA, *P* < .001, Figure 6, C and D, right panel). There was no change in tumor size of mice treated with EGFP-ADSCs (*P* = .216) or TRAIL-ADSCs (*P* = .907, Figure 6, C and D, right panel). We examined whether genetically modified ADSC (GM-ADSC) treatments improved the overall survival of mice with melanoma lung metastases. Treatment of mice with IFNγ-ADSCs or IFNγ/TRAIL-ADSCs demonstrated a statistically significant increase in the median survival of melanoma bearing mice, while most of the animals in other groups died by day 40 (Figure 7A). Results from the Pairwise log-rank test indicated there was no difference between survival of mice treated with IFNγ-ADSCs or IFNγ/TRAIL-ADSCs (Additional file 1: Table S3). Results from immunohistochemistry (IHC) and TUNEL staining shown in Figure 7, B and C indicated a statistically significant decrease in melanoma cell proliferation (Ki67; *P* < .001), tumor vasculature (CD31; *P* < .001) and a statistically significant increase in melanoma cell apoptosis (TUNEL; *P* < .001). Immunostaining of pulmonary tumor sections revealed that injected IFNγ-producing ADSCs upregulate PD-L1 expression in melanoma lung tumor cells (compared to metastatic tumor samples from mice that received EGFP-ADSCs or remained untreated) (Figure 7B). Differentiation staining indicated there was no *in vivo* differentiation of ADSCs from different groups into three mesodermal lineages in tumor samples (Additional file 2: Figure S1, available online). However, some capillary-like structures of ADSCs were observed after IHC for EGFP and PD-L1 (Additional file 3: Figure S2, available online).

### Murine ADSC homing

**Figure 8:**
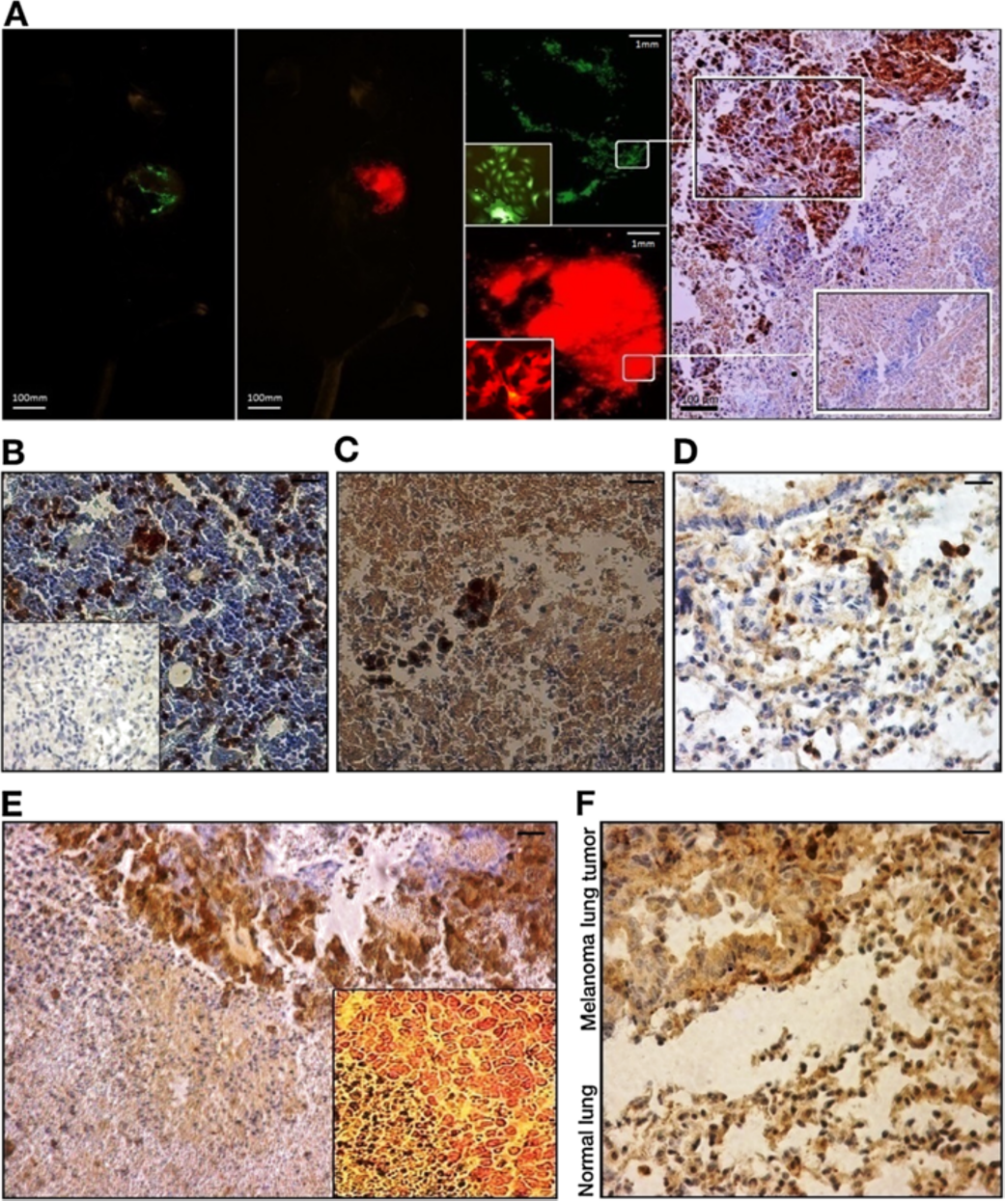
**Homing analysis of ADSCs for EGFP and IFNγ in subcutaneous tumor and lung metastatic models. A)** Representative *In vivo* imaging of green and red fluorescence optical imaging in mouse with subcutaneously co-injected EGFP-ADSCs and RFP-melanoma cells. Focal imaging indicates aggregation of EGFP-expressing ADSCs around the melanoma cells in the tumor. IHC results of subcutaneous tumor sections for EGFP indicate that melanoma cells and ADSCs are not mixed completely in the tumor. **B** and **C)** IHC analysis of EGFP + cells. Scale bars =25 μm. Panel **B** indicates a representative microscopic image of staining for a section of the bone marrow derived from an EGFP transgenic mouse as a positive control. Inlay shows a representative image of staining for a section of melanoma lung tumor from control group. Panel **C** shows a representative microscopic image of a melanoma lung tumor section after injection of EGFP-ADSCs. **D**, **E**, and **F)** IHC analysis of IFNγ + cells. Panel **D)** demonstrates a representative microscopic image of a melanoma lung tumor section after injection of IFNγ-ADSCs. Scale bar =25 μm. Panel **E)** depicts a representative microscopic image of staining for a section of subcutaneous melanoma tumor which indicates juxtaposition of IFNγ-ADSCs with melanoma cells. Scale bar =50 μm. The Inlay shows representative image of TUNEL staining for IFNγ-ADSC/melanoma mixed tumor samples which also confirm position of IFNγ-ADSCs alongside the melanoma cells. Panel **F)** Representative image which demonstrates both melanoma lung tissue and normal lung tissue. The image indicates that most of IFNγ-ADSCs homed into melanoma lung tissue rather than normal lung tissue. Scale bar =50 μm.

To evaluate the homing behavior of EGFP-ADSCs, which had been co-injected with RFP-melanoma cells, subcutaneous tumor bearing mice were analyzed 21 days post intravenous injection using a In-Vivo imaging system. We detected EGFP-ADSCs accumulating around the tumors (Figure 8A), a finding confirmed by IHC analysis of subcutaneous tumor sections (Figure 8A). Likewise, the IHC analysis for IFNγ and TUNEL staining showed juxtaposition of IFNγ-ADSCs with melanoma cells (Figure 8E). We also tracked homing of EGFP-ADSCs and IFNγ-ADSCs to pulmonary B16F10 tumors. Melanoma nodules were harvested at day 28 post melanoma cell injection. The IHC analysis of melanoma lung tumor sections using a polyclonal anti-EGFP Ab indicated homing of ~4.3 of EGFP-positive cells to the melanoma metastases in the lung (Figure 8, B and C). IHC analysis of melanoma metastases using a monoclonal anti-mouse IFNγ Ab showed ~4.7 of IFNγ-positive cells per 1000 melanoma tumor cells (Figure 8D). Detection of IFNγ in the melanoma lung tumor samples confirmed prolonged expression of IFNγ by IFNγ-ADSCs, most of which homed to the melanoma lung metastases and not to normal lung tissue (Figure 8F).

### The effect of cell therapy on infiltration of CD4+, CD8+ ,FOXP3+ and IL2+ Cells

**Figure 9:**
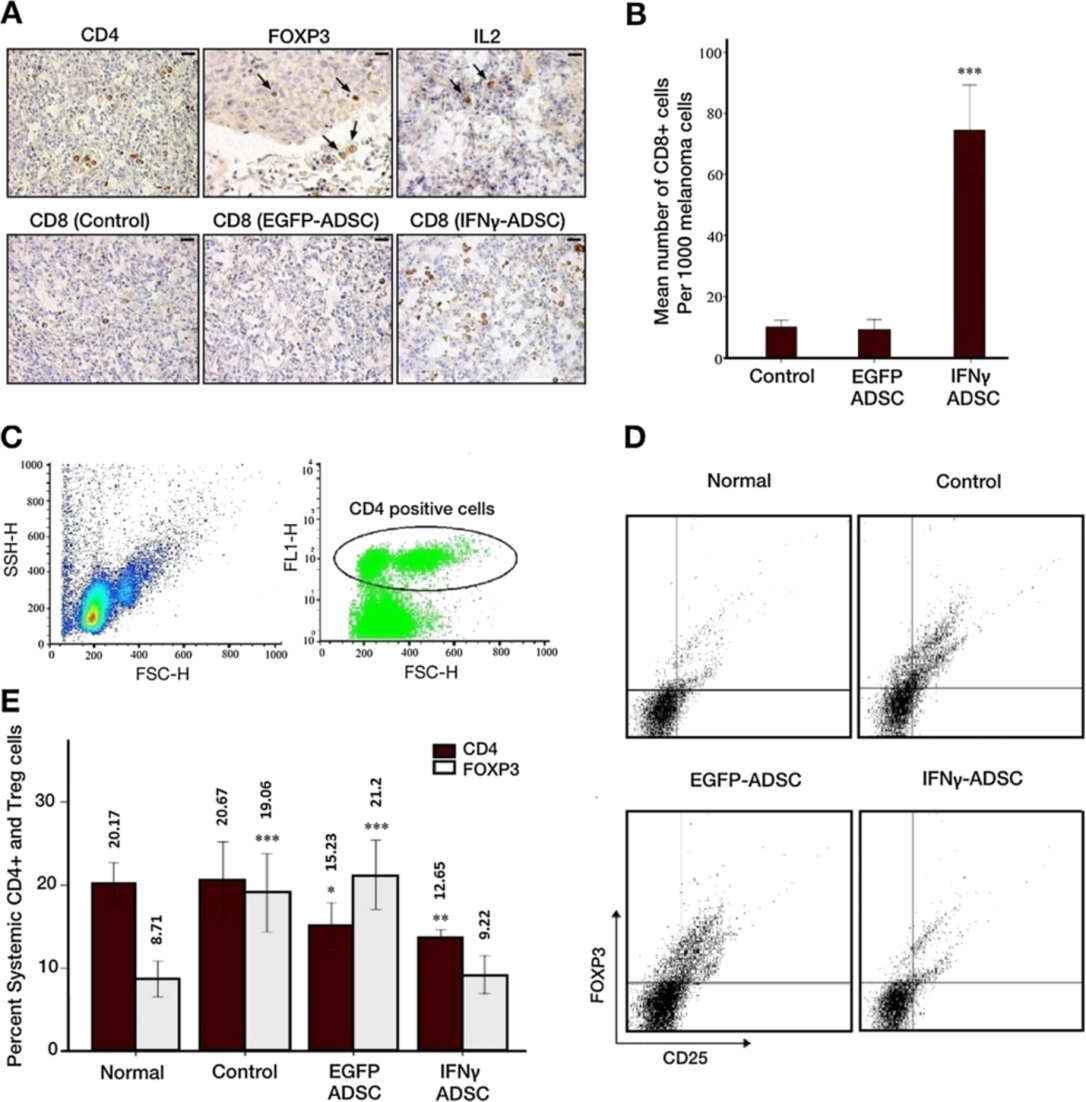
**Determination of immune effectors following stem cell therapy. A)** Infiltration of immune cells into the melanoma lung tumors. The lung tumors stained with monoclonal antibodies specific for mouse CD4, FOXP3, IL2 and CD8. Only respective images of CD4+, FOXP3+ and IL2+ cells from control samples are shown as there was no statistically significant difference between groups. Black arrows denote FOXP3+ and Il2+ cells. Results show significant infiltration of CD8+ cell into the melanoma lung tumors after injection of IFNγ-expressing ADSCs. Scale bars =50 μm. **B)** As shown in the bar graphs, mean number of CD8+ cells significantly increased in metastatic melanoma tumors of mice treated with IFNγ-ADSCs in a statistically meaningful manner. N =6; ***P < .001. **C** and **D)** Representative FACS plots for analysis of systemic CD4+ and regulatory T cells in peripheral blood obtained from the sub-ocular region of mice with established melanoma metastases. In addition, blood samples were taken from normal mice to measure normal number of CD4+ and Tregs. **C)** Left plot is representative FACS plot showing two distinct populations of PBMCs: lymphocytes and monocytes. Right plot is representative FACS plot indicating CD4 positive PBMCs. **D)** CD4+ lymphocytes were analyzed for expression of CD25 and FOXP3 by flow cytometry. Representative plots indicating analysis of Tregs in Normal, Control, EGFP-ADSCs and IFNγ-ADSCs. **E)** The percentage of CD4+ cells indicated a statistically significant decreased systemic level of CD4+ cells in ADSC-injected groups. The plot also shows a statistically significant decrease in the percentage of Tregs in the peripheral blood of mice with melanoma metastases treated with IFNγ-producing ADSCs. This percentage is comparable with systemic regulatory T cells of normal mice (*P* = .109; IFNγ-ADSCs compared to normal mice). N =6; ***P < .001, **P < .01, *P < .05.

We examined the infiltration of CD4+, CD8+, FOXP3+ and IL2+ cells to evaluate a potential participation of immune cells in GM-ADSC therapy experiments. An IHC analysis of metastatic lung tumors was performed at day 28 of the stem cell treatment. Metastatic lung tumors treated by IFNγ revealed no statistically significant infiltration of CD4+, FOXP3+ and IL2+ cells compared to control group (Figure 9A, upper panel, respective microscopic images from control samples are shown), whereas there was a statistically significant increase in CD8+ cells (ANOVA; *P* < .001, Figure 9B), indicating IFNγ-induced infiltration of CD8+ T cells (Figure 9A, lower panel).

### The effect of cell therapy on population of systemic CD4^+^CD25^+^FOXP3^+^ T regulatory cells (Tregs)

Population analysis of peripheral blood mononuclear cell (PBMC) isolated by Ficoll density gradient revealed two separate populations of PBMCs; lymphocytes and monocytes (Figure 9C, left panel). We initially analyzed PBMCs and lymphocytes for CD4 (Figure 9C, right panel) and subsequently CD4+ lymphocytes for CD25^+^ FOXP3^+^ by FACS analysis (Figure 9D). We noted that the mean percentage of CD4+ cells significantly decreased in ADSC-injected groups in a statistically meaningful manner (ANOVA, *P* = .021 for EGFP-ADSCs and *P* =0.002 for IFNγ-ADCS compared to normal mice, Figure 9E). Moreover, our results indicated that the population of Tregs in mice treated with IFNγ-ADCS (*P* =0.109) and IFNγ/TRAIL-ADSCs (*P* =0.797) were comparable to the population of Tregs in normal mice. In contrast, the population of Tregs were increased significantly in a statistical manner in mice with established B16F10 lung metastases, which were injected with PBS only, EGFP-ADSCs or TRAIL-ADSCs (ANOVA, *P* < .001, Figure 9E).

## Discussion

In the present study, we demonstrated that *φC31* and/or pB*t* mediated non-viral gene transfer provides prolonged and high levels of transgene expression in ADSCs. Both the recombinase and the transposase systems worked well for modification of ADSCs. However, genomic modifications mediated by *φC31* may result in chromosomal rearrangements [21], whereas pB*t* is deactivated during the excision of the transposon from the helper-independent p*mhy*GENIE-3 construct [6]. We observed robust levels of IFNγ and TRAIL expression in GM-ADSCs conferring upon them the ability to be efficiently used for cancer therapy. We also demonstrated that exogenously administered ADSCs survive and proliferate in the tumor environment. When co-injected with melanoma cells, ADSCs mostly stayed along with melanoma cells. We explored the effect of TRAIL-ADSCs and we showed that murine TRAIL, with a homology of 65% to human TRAIL is active on human CAOV-4 and Ej-138 cell lines [22]. However, certain tumor cells such as melanoma B16F10 and human MCF-7 breast cancer cell lines used in this study are not amenable to TRAIL-mediated apoptosis [23]. IFNγ may sensitize cancer cells to TRAIL-induced apoptosis by upregulating Caspase-8 through a Stat1/IRF1 dependent pathway [11–16]. Still, we did not observe sensitization of melanoma cells to TRAIL in the presence of IFNγ. Therefore, melanoma resistance is probably based upon lower expression of functional death receptors (DR1 and DR2) [24]. Actually, a recent study suggests the role of NFκB-mediated inflammatory signals through the death receptor DR5 which may promote malignant behaviors of melanoma cells [24]. Interestingly, the effect of IFNγ on melanoma B16F10 cells was remarkable. IFNγ has been clinically applied to treat a variety of malignancies [8]. Phase I trials of IFNγ gene transfer into cancer cells in patients with metastatic melanoma have been performed, albeit with low therapeutic efficacy [25–27]. Furthermore, it has been shown that human IFNγ produced by genetically modified MSCs was able to inhibit proliferation and induce apoptosis of human leukemia K562 cells *in vitro* [28]. We demonstrated that IFNγ secreted by GM-ADSCs is able to directly affect melanoma cells *in vitro,* probably through activation of JAk1/Stat1 pathway [29]. Moreover, co-injection of IFNγ-ADSCs with melanoma cells reduced the growth of subcutaneous melanoma tumors. More importantly, intravenously injected IFNγ-ADSCs significantly reduced growth of pre-established melanoma lung metastases. In addition, systemic delivery of IFNγ-ADSCs does not elevate IFNγ amounts in blood circulation, suggesting local expression of IFNγ. We also explored the anti-tumor effects of IFNγ through the activation of the immune response [30]. We noted a significantly increased infiltration of CD8+ cells, while the CD4+ and IL2+ cell count in the tumor environment of IFNγ-ADSC treated mice remained constant. Additionally, we confirmed IFNγ-induced overexpression of PD-L1 in melanoma tumor cells [31, 32]. These observations suggest a possible role of PD-L1 in impairing T cell function because of; 1) the absence of infiltrated T helper 1 cells (no statistically significant difference in CD4+ and IL2+ cells) which is critical for the development of cell-mediated immune response [32], and 2) the accumulation of exhausted and inflamed CD8+ cells which failed to produce IL2. The latter could explain why melanoma metastases avoided complete eradication in spite of significant infiltration of CD8+ cells [33]. Actually, CD8+ T cells and IFNγ may induce the expression of indoleamine-2,3-dioxygenase (IDO) and PD-L1 in the melanoma tumor microenvironment, and inhibit the activation of additional T cells trafficking [34]. Therefore, we hypothesize that the observed anti-tumor effect was primarily due to apoptosis in the melanoma tumor and tumor endothelial cells. A similar observation has been reported by Kakuta et al., where they examined effects of IFNγ receptor-deficiency and NK cell depletion on the melanoma tumor growth and suggested that IFNγ prevents the murine melanoma metastases by directly inhibiting cell growth when the tumor mass is small and in an earlier developmental stage [35]. It is also noteworthy that the population of infiltrated FOXP3+ cells in IFNγ-ADSC treated mice did not differ to those in mice of other treatment groups, indicating that the higher systemic Tregs (in control and EGFP-ADSC injected mice) was possibly due to immunosuppression mediated by tumor progression [36]. Therefore, intravenous injection of IFNγ-ADSC indirectly maintained normal levels of systemic Treg through inhibition of melanoma growth [36]. However, systemic injection of GM-ADSCs showed immunosuppressive properties probably associated with PD-L1 expression by ADSCs [37]. The selective blockage of PD-L1 may be a potential strategy to improve the therapeutic value of IFNγ-producing ADSCs for melanoma treatment [38].

We were able to show that systematically transplanted ADSCs engrafted into tumor cells, however the fact that stem cells also migrate to many other organs makes it difficult to trace them. In fact, there is no clear consensus amongst researchers where else such treated stem cells end up after injection [39]. It is well known that systemically infused MSCs typically become trapped within the lungs as the first micro capillary network they encounter (pulmonary "first-pass" effect) [40]. Within 24h MSCs migrate to other organs, in particular the liver and also the spleen [41, 42]. Consecutively, MSCs appear at injured tissue sites and tumors as well as bone marrow, liver and spleen [43, 44]. This redistribution attributes to the observed reduction of MSCs present in the lungs, as does cell death [45]. Kidd et al. showed that in addition to the specific tropism of MSCs in tumor sites, some MSCs remained in the lungs, as well as some disseminating into the liver [46]. An alternate study showed that few days after tail vein stem cell injections, cells dissipated from the animals and were undetectable within one week after injection [47].

Studies have attempted to address the eventual fate of the MSCs within tumor microenvironments. MSC may differentiate towards tumor-associated fibroblast (TAF) phenotypes [48]. In addition, MSCs may acquire endothelial-like characteristics, but their involvement in vasculogenesis is complex. Comşa et al. indicated that MSCs, negative for CD31, have a clear tendency to form capillary-like structures in the presence of tumor-derived VEGF [49]. The same proved true in our hands where ADSCs co-injected with melanoma cells organized into capillary-like structures, whereas, they did not differentiate into three mesodermal lineages in the melanoma tumor environment.

In order to translate stem cell-based anticancer strategies into clinical therapy, it is essential to identify and minimize treatment-associated risks. Only with improvements in safety, quality, and efficiency of stem cell/gene therapy for inoperable or malignant tumors, clinical scenarios can be envisaged. For the first time we employed DNA integrating vectors including *PhiC31and PiggyBac* transposase systems for safe and stable modification of MSCs. We modified ADSCs to produce IFNγ and TRAIL. Then evaluated antitumor effects of cytokine-producing GM-ADSCs in murine models of melanoma. The present study is the first *in vivo* attempt to use ADSCs as a vehicle for IFNγ-mediated immunotherapy and demonstrates the potential of non-virally modified IFNγ-ADSCs for melanoma cancer therapy. This may have a significant role in the management of cancer in the future.

## Materials and methods

### Isolation of ADSCs and culture

ADSCs were isolated from inguinal fat pads of 4–5 week-old male C57BL6 mice (N = 8; 21.5 ± 0.7 g body weight). Isolated cells were grown in culture medium (CM) consisting of Dulbecco’s modified Eagle’s medium (DMEM; Invitrogen Carlsbad, CA), 10% Fetal bovine serum (FBS; Invitrogen Carlsbad, CA), 1 g/L glucose, 1% L-glutamine, and 1% penicillin-streptomycin (Invitrogen Carlsbad, CA). For details please see Additional file 1.

### ADSCs differentiation

ADSCs cultured at passage 6 were used for adipogenic, osteogenic, and chondrogenic differentiation. As a first step, cells were cultured for 3 weeks in differentiation medium and consecutively stained with Oil red O, Alizarin red S, Von Kossa S, Alcian blue, and Toluidine blue. For a detailed description of the experiments please see Additional file 1.

### Immunotyping of ADSCs

Immunotyping of surface markers was performed in passage 6 with BD Calibur^™^ (Becton Dickinson San Jose, CA) using monoclonal antibodies against mouse CD11b, CD24, CD34, CD45, CD73, CD90.1, CD105, CD133, CD146, CD309 and CXCR4 (all form eBioscience, San Diego, CA). A total of 5 × 10^5^ cells from passage 6 were incubated with each antibody (Ab) in PBS with 3% bovine serum albumin (BSA; Sigma, St. Louis, MO) for 40 min at 4°C. The Cells were then washed with PBS and fixed with BD fixation reagent (BD Biosciences, San Jose, CA sciences). Analysis of the FACS data was carried out with a FlowJo software version 10 (Treestar, OR).

### Vector construction

Plasmids pCAG-DsReds [21], pCMVInt [6], pDRBB2 [50], pBEB [6], pISRE-TA-Luc (Clontech, Mountain View, CA), pBLB [6], p*mhy*GENIE-3 [51], coding sequences (CDS) of murine full-length IFNγ, TRAIL and the sequence of SV40 Promoter were used in this study. The plasmid pBEB (carrying the *attB*, the enhanced green fluorescent protein [EGFP] reporter gene) and the vector p*mhy*GENIE-3 (containing EGFP gene, a hyperactive self inactivating *piggyBac* sequence and *piggyBac* transposon) were used as a control plasmids for integrase and transposase systems. We prepared the vector pDsRed-attb-zeo (carrying eRFP, *attB,* eukaryotic zeocin resistance gene) using the plasmid pCAG-DsReds (expressing the Red fluorescent protein [eRFP] reporter gene), the plasmid pDRBB2 (carrying *attB* and antibiotic resistance gene for zeocin under the prokaryotic T7 promoter) and a synthesized sequence of T7 promoter. The vector pBTB (carrying *attB* and antibiotic resistance gene for G418 and a murine full-length TRAIL) was prepared using the vector pBLB (carrying the *attB* and firefly luciferase [FL] under a chicken beta actin and rabbit beta globin intron [CAG] promoter) and synthesized murine TRAIL coding sequence (CDS; protein ID = NP_033451.1). The plasmid pISRE-TA-Luc-attB was prepared using the vector pISRE-TA-Luc (carrying FL gene located downstream of ISRE enhancer element and a minimal TA promoter; Clontech, Palo Alto CA) and pBLB. All integrase-related constructs were co-transfected with the plasmid pCMVInt (producing *φC31* integrase) to achieve stable cell modification. Plasmids pDsRed-attb-zeo, p*mhy*GENIE-3 and the coding sequence of the full-length murine IFNγ (protein ID = NP_032363.1) were used for construction of p*mhy*GENIE-3-IFNγ. CDS of murine full-length IFNγ, TRAIL and the sequence of SV40 Promoter with pertinent restriction sites were synthesized by Generay Biotech (Co., Ltd). All vectors were purified by EndoFree® plasmid maxi kit (Qiagen, Valencia, CA) before transfection. For additional information on vector construction please see Additional file 1.

### Murine ADSCs transfection, selection, and transgene expression

Nucleofection was perfomed by nucleofector device 2b (Amaxa Biosystems) using the Human MSC Nucleofector kit (Amaxa Biosystems) with program X-001 (mouse T cells). About 2 μg of DNA plasmid and 5 × 10^5^ ADSCs were used for each nucleofection experiment and done in triplicate for each plasmid construct. The vector pMAX was utilized for evaluation of nucleofection efficiency. ADSCs modified with pBEB/pCMVInt, pBIB/pCMVInt, p*mhy*GENIE-3, and p*mhy*GENIE-3-IFNγ are referred to as EGFP/Int-ADSC, TRAIL-ADSC, EGFP-ADSC and IFNγ-ADSC respectively. TRAIL-ADSCs were modified with p*mhy*GENIE-3-IFNγ to generate ADSCs co-expressing TRAIL and IFNγ (referred as IFNγ/TRAIL-ADSC). After each nucleofection, 500 μl of CM was added to each nucleofector cuvette and cells were seeded into 6 well plates at 37°C in 5% CO_2_. Forty eight hours after each nucleofection, selection with related resistant antibiotic was initiated. ADSCs co-nucleofected with the vectors pBEB/pCMVInt (EGFP/Int-ADSC) and pBTB/pCMVInt (TRAIL-ADSC) were exposed to 1000 μg/mL of G418 sulfate (Roche, Indianapolis, IA) for two weeks and maintained in CM with 800 μg/mL of G418 sulfate. ADSCs nucleofected with the plasmids pmhyGENIE-3 (GFP-ADSC) and p*mhy*GENIE-3-IFNγ (IFNγ-ADSC) were selected with 200 μg/mL of hygromycin (Roche, Indianapolis, IA) for about 10 days and maintained under selection pressure with 100 μg/mL of hygromycin, giving rise to EGFP-ADSCs and IFNγ-ADSCs. After about 2 weeks 15 × 10^4^ of TRAIL-ADSCs were nucleofected with p*mhy*GENIE-3-IFNγ and selected with hygromycin to create TRAIL/IFNγ co-expressing ADSCs (TRAIL/IFNγ-ADSCs). These cells were maintained in a CM containing G418 (800 μg/mL) and hygromycin (100 μg/mL). Surface and intracellular staining of TRAIL-ADSCs and EGFP/Int-ADSC (control) were done with anti-TRAIL Ab (eBioscience, San Diego, CA). Expression of IFNγ was confirmed by Western blot. Protein extraction was performed by lysing EGFP-ADSCs and IFNγ-ADSCs with Cell lysis buffer (Sigma, St. Louis, MO) supplemented with a protease inhibitor cocktail (100 μg/mL; Sigma, St. Louis, MO). Proteins were separated in 12% SDS PAGE and transferred to nitrocellulose membrane (Amersham Biosciences/GE Healthcare, Pittsburgh, PA). The membrane was blocked in buffer containing PBS, 10% powdered nonfat milk (Sigma, St. Louis, MO), and 0.05% Tween-20 (Sigma, St. Louis, MO). Blotting was performed with a monoclonal rabbit anti-mouse IFNγ Ab at dilution of 1:1000. After twice washing the membrane with blocking buffer, blotting continued using a horseradish peroxidase (HRP)-conjugated anti-rabbit secondary antibody at 1:1000 dilution (Abcam, Cambridge, MA). Interferon-γ protein band was detected after adding the enhanced chemiluminescence reagent (Amersham Biosciences/GE Healthcare, Little Chalfont) by an imaging system (Bruker Inc, Ettlingen, Germany) using UV-Epi-Illumination source with 30 seconds of exposure time.

### Cancer cell culture, transfection, and selection

The mouse melanoma cell line B16F10, human breast cancer cell line MCF-7, human bladder carcinoma derived cell line Ej-138, and human ovarian carcinoma cell line CAOV-4 were purchased from the Pasteur institute of Iran. Cancer cells were maintained in CM at 37°C in a 5% CO_2_ atmosphere. In this study, melanoma cells which were modified with pBEB/pCMVInt and pDsRed-attb-zeo/pCMVInt vectors are referred to GFP-melanoma and RFP-melanoma cells respectively. Lipofection was used for modification of melanoma cells. To create transgenic melanoma B16F10, cells at a density of 2 × 10^5^ per well in triplicate for each group were seeded in 6-well plates. After 24 h, cells were co-transfected with the vector pBEB/pCMVInt, pBLB/pCMVInt, pISRE-TA-LUC-attb/pCMVInt, and pDsRed-attb-zeo/pCMVInt using lipofectamine 2000^™^ transfection reagent (Invitrogen, Carlsbad, CA) according to the manufacturer’s instructions. EGFP-melanoma, FL-melanoma and ISRE/FL-melanoma were selected, and maintained with 1000 μg/mL of G418. RFP-melanoma was exposed to 400 μg/mL zeocin (Invitrogen, Carlsbad, CA) and after about two weeks maintained in CM with 100 μg/mL of zeocin.

### Bioactivity of IFNγ and TRAIL produced by ADSCs

To study the effect of IFNγ-ADSC and TRAIL-ADSC on melanoma B16F10 cells we evaluated proliferation and apoptosis by MTT (Sigma, St. Louis, MO) and Annexin V apoptosis (Roche, Indianapolis, IA) assays. MTT and Annexin V apoptosis assays were performed as explained in Additional file 1.

### Monitoring the induction of the STAT1/2 components of Jak/STAT-mediated signal transduction pathways

FL-melanoma and ISRE/FL-melanoma cells were seeded into 12 well plates and incubated at 37°C in 5% CO_2_. Supernatants from either EGFP-ADSCs or IFNγ-ADSCs were added to these cells once they reached cell densities of 10^5^ and 3 × 10^5^ respectively. Two days later, a volume of ONE-Glo™ luciferase assay reagent (Promega Corp. Madison, WI) equal to that of the CM was added to each well and samples were mixed thoroughly. Luminescence imaging was performed in an imaging system (Bruker Inc, Ettlingen, Germany) using a UV-Epi-Illumination source with a 15 min exposure time.

### *In vivo*studies and optical imaging

Eight-week-old male C57BL/6 mice (30.1 ± 0.6 g body weight) were purchased from the Pasteur institute of Iran. Handling of the animals was performed according to the guidelines of the Institutional Animal Care and Ethics Committee of Isfahan University. For the subcutaneous models of melanoma, 2 × 10^6^ RFP-melanoma cells were delivered either alone or concurrently with 7.5 × 10^5^ ADSCs of each group described above, in 200 μl of PBS into the right flank of the mice. The day of melanoma injection was defined as day 0. At day 21, *in vivo* red fluorescence optical imaging of subcutaneous RFP-melanoma bearing mice was performed. Subsequently, all mice were euthanized via pentobarbital overdose. In lung metastatic models, 7.5 × 10^5^ RFP-melanoma cells were delivered into the lateral tail vein of twelve mice per group. Ten days later, the mice received 7.5 × 10^5^ of GM-ADSC in a volume of 200 μl of PBS solution (control group received only 200 μl of PBS) by tail vein injection. Six mice from each group were sacrificed with pentobarbital overdose at day-28. Subsequently, their lungs were dissected and *ex vivo* red fluorescence optical imaging of lung samples was performed. The six remaining mice from each group were kept alive for long-term survival analysis. For imaging, animals were anesthetized with 2% isoflurane and images were captured using a multi-wavelength source with a 2 min exposure time in the In-Vivo F Pro small-animal imaging system (Bruker Inc, Ettlingen, Germany).

### Serum levels of IFNγ

Frozen serum collected from blood samples at days 10, 16, and 21 was used in triplicate to quantify IFNγ protein levels by enzyme-linked immunosorbent assay (ELISA) using the mouse IFNγ Elipair kit (Abcam, Cambridge, MA) in accordance with the manufacturer’s protocol. Samples were analyzed at an absorbance of 450 nm with minimal sensitivity of 15 pg/mL for IFNγ detection.

### Analysis of systemic CD4+ and CD4^+^CD25^+^FOXP3^+^ regulatory T cells (Treg)

Peripheral blood was taken from sub-ocular regions of normal C57BL/6 mice (control samples) and lung metastatic melanoma bearing mice at day 28 post melanoma cell injection. Blood was overlaid onto 3 mL of Ficoll-Paque and after centrifuging at 1800 rpm for 15 minutes, peripheral blood mononuclear cells (PBMCs) at the interface were collected. PBMCs were washed twice in PBS and then Treg cells were analyzed using a mouse Treg detection kit (Miltenyi Biotec, Auburn, CA). PBMCs were stained with FITC-conjugated monoclonal anti-mouse CD4 Ab and APC-conjugated monoclonal anti-mouse CD25 Ab for 30 minutes at 4°C in the dark. Subsequently, the PBMCs were incubated with a PE-conjugated monoclonal anti-mouse Foxp3 Ab, following the staining protocol provided by Miltenyi Biotec. FACS data analysis was performed with BD CellQuest Pro or FlowJo software.

### Histological analysis

Tissue sections were used for differentiation staining, terminal deoxynucleotidyl transferase-mediated dUTP-biotin nick end labeling (TUNEL) staining and immunohistochemistry (IHC) analysis. IHC was performed for EGFP, Ki67, murine IFNγ, murine IL2, programmed cell death 1 ligand 1 (PD-L1; B7-H1), CD31, CD4+, CD8+ and FOXP3+. For a detailed description of the experiments please see Additional file 1.

### Statistical analysis

Statistical analysis was performed using SPSS version 19 (SPSS Inc, Chicago, IL). The statistical differences between the groups were assessed by Student t test, ANOVA, and log-rank tests. Survival was defined as the date of melanoma cell injection to the date of death. *P* values less than .05 were considered statistically significant. All statistical tests were two-sided. Data are presented as mean values with 95% confidence intervals (CIs).

## Electronic supplementary material

Additional file 1: **Supplementary data: Supplementary Methods, Reference and Tables (** **Table S1**, **Table S2** **and** **Table S3).** (DOC 72 KB)

Additional file 2: Figure S1: Differentiation analysis of melanoma tissues. Mouse fat tissue from the inguinal fat pad, murine cartilage isolated from patellae and mouse bone tissue obtained from a 8 week-old male C57BL6 mouse were used as positive controls (Left panels). Representative images from melanoma/IFNγ-ADSCs co-injected groups are shown (Right panels). Results indicated there was no *in vivo* differentiation of ADSCs from different groups into three mesodermal lineages. Scale bars = 50 μm. A) Oil red O staining. B) Alician blue staining. C) Alizarin red staining. ADSC = adipose derived mesenchymal stem cell, IFNγ = interferon gamma. (JPEG 324 KB)

Additional file 3: Figure S2: Formation of capillary-like structures by ADSCs in ADSCs/Melanoma co-injected groups. A) Representative microscopic images of immunohistochemistry (IHC) staining for EGFP. (Left) Melanoma/PBS (Right) Melanoma/EGFP. Capillary-like structures are formed by EGFP expressing ADSCs. B) Representative microscopic images of IHC staining for PD-L1. (Left) Melanoma/PBS (Right) Melanoma/EGFP. Mesenchymal stem cells highly expressing PD-L1 have organized capillary-like structures. (JPEG 523 KB)
