# Supplementary material for: PhiC31/PiggyBac modified stromal stem cells: effect of interferon γ and/or tumor necrosis factor (TNF)-related apoptosis-inducing ligand (TRAIL) on murine melanoma
Source: Mol Cancer. 2014 Nov 26;13:255. doi: 10.1186/1476-4598-13-255 (PMC4258801; doi:10.1186/1476-4598-13-255)
Supplement: Supplementary file 1 — Additional file 1: Supplementary data: Supplementary Methods, Reference and Tables ( Table S1, Table S2 and Table S3). (DOC 72 KB) [file 12943_2014_1455_MOESM1_ESM.doc]

**Supplementary Methods**

**Isolation of ADSCs and Culture**

Tissues were isolated from inguinal fat pads, rinsed with phosphate buffered saline (PBS; Invitrogen Carlsbad, CA) and treated with 0.075% collagenase type I (Sigma, St. Louis, MO) at 37°C for 45 minutes with a manual agitation every 10 min. The digestion process was stopped with equal volume of culture medium (CM). The cellular content were collected after Centrifugation at 1400 rpm for 10 min. Cells were then washed and cultured in CM supplemented with 0.5 mg/mL amphotericin B (Gibco-BRL) at 37˚C in 5% CO_2_. Isolated ADSCs were grown in CM supplemented with 1 ng/mL fibroblast growth factor (FGF; Sigma, St. Louis, MO), and 10 ng/mL epidermal growth factor (EGF; Sigma, St. Louis, MO) to maintain undifferentiated state of ADSCs (1).

**ADSCs Differentiation**

ADSCs were subsequently used for adipogenic, osteogenic and chondrogenic differentiation. For adipogenic differentiation, 2.5×10^4^ ADSCs were treated with CM supplemented with 1% Insulin-Transferin-Selenium (ITS; Invitrogen Carlsbad, CA), 250 nM dexamethasone (Sigma, St. Louis, MO) and .5 mM 3-isobutyl-l-methylxanthine (IBMX; Sigma, St. Louis, MO) for 12 days. Differentiated cells were then fixed with 4% paraformaldehyde and stained with 0.5% Oil red O (Sigma, St. Louis, MO) for 10 min in room temperature (RT). To initiate osteogenesis, 2.5×10^4^ ADSCs were cultured in differentiation medium consisting of 10 nM beta glycerol-phosphate (Merck, Darmstadt), 50 µg/mL ascorbic acid 2 phosphate (Sigma, St. Louis, MO), 100 nM dexamethasone, and 10^−8^ M 1α 25-dihydroxyvitamin D3 (1,25(OH)2D3; Calbiochem, Billerica, MA) for 3 weeks. The cells were then fixed with 4% paraformaldehyde and stained with Alizarin red (Sigma, St. Louis, MO) and Von Kosa Staining. To induce cartilage differentiation, 2.5×10^4^ ADSCs were centrifuged at 1400 rpm. Cell pellets were treated with CM containing 50 µg/mL ascorbic acid 2 phosphate, 10 ng/mL transforming growth factor β (TGF-β; PeproTech, Rocky Hill, NJ) and 100 nMdexamethasone for 21 days. Cells were fixed with 4% paraformaldehyde and chondrogenic differentiation was visualized by staining paraffin embedded sections with Toluidine blue (Sigma, St. Louis, MO) and Alician blue (Sigma, St. Louis, MO).

**Vector Construction**

For construction of the vector pDsRed-attb-zeo, the plasmid pDRBB2 was double digested with *Sac*I/*Bgl*II restriction enzymes (RE) (Thermo Fisher Scientific, Waltham, MA). The larger gel extracted fragment (~ 4.6kb) was blunt ended using T4 DNA polymerase (Thermo Fisher Scientific, Waltham, MA) and dephosphorylated with calf alkaline phosphatase (Takara). In parallel, pCAG-DsReds was excised with HindIII and ScaI RE (Thermo Fisher Scientific, Waltham, MA) and the larger gel isolated fragment (~3.4kb) was blunt ended using T4 DNA polymerase and ligated into the larger fragment of pDRBB2 vector. The resulting product was a vector containing the attb, the RFP cassette, and an antibiotic resistance gene for zeocin under the control of the T7 promoter. To insert a eukaryotic promoter for the zeocin resistance gene, the above described vector was linearized with HindIII RE, followed by calf alkaline phosphatase dephosphorylation. A SV40 promoter was excised from the plasmid pGH with HindIII RE and ligated into the linearized, dephosphorylated vector described above. To create the vector pBTB, plasmid pBLB was digested with XbaI RE (Thermo Fisher Scientific, Waltham, MA) and the luciferase gene was replaced with murine TRAIL CDS. During the construction of the vector pISRE-TA-LUC-attb, plasmids pBLB and pISRE-TA-Luc were excised with AseI and XbaI RE (Thermo Fisher Scientific, Waltham, MA) and the FL gene cassette in the vector pBLB was replaced with the FL gene under the ISRE enhancer element and the minimal TA promoter. To construct pmhyGENIE-3-IFNγ, the vector pmhyGENIE-3 was RE digested with NheI (Thermo Fisher Scientific, Waltham, MA) and HpaI (NEB; England BioLabs, Beverly, MA) and used as a backbone for the incorporation of IFNγ cassette. In parallel, the RFP gene from the vector pDsRed-attb-zeo was removed following AgeI/NotI RE double digestion (Promega Corp. Madison, WI) and IFNγ CDS was cloned into its position after ligation with T4 DNA ligase. The resulting product was digested with SpeI RE (Thermo Fisher Scientific, Waltham, MA) and StuI RE (England BioLabs, Beverly, MA) and the smaller fragment carrying the IFNγ cassette was ligated into linearized pmhyGENIE-3 vector (compatible end sites of NheI/SpeI and HpaI/StuI).

**Bioactivity of IFNγ and TRAIL Produced by ADSCs**

For the MTT assay, melanoma cells at a density of 7.5 ×10^3^ cells per well were seeded in 96 well plates (Orange Scientific, Braine-L’Alleud, Belgium), and incubated at 37˚C in 5% CO_2_ for 24h. CM of cells was replaced with either CM from EGFP-ADSCs or IFNγ-ADSCs (48h after CM replacement of ADSCs without selection antibiotic). Zeocin was used as a positive control (10^4^ μg/mL) and recombinant mouse IFNγ (Abcam, Cambridge) was used as a sample control. Plates were returned to the incubator for 68h. MTT dye was added to each well and after 4h the reaction was stopped with the addition of dimethyl sulfoxide (DMSO; Sigma, St. Louis, MO) and the optical density was determined at 570 nm in a multiwell plate reader (model ELX800; Bio-kit). The potential of IFNγ secreted from ADSCs to induce apoptosis in melanoma B16F10 was evaluated by Annexin V apoptosis assay. Melanoma cells at a density of 10^5^ were seeded into 12 well plates (four groups in triplicate) at 37˚C in 5% CO_2_. After 24h melanoma supernatants were replaced with supernatants from either EGFP-ADSCs or IFNγ-ADSCs (CM without hygromycin). The melanoma cells were then returned to the incubator. After 12h and 24h melanoma cells were stained with Annexin-V-FLUOS Staining Kit (Roche, Indianapolis, IA). To assay apoptotic potential of TRAIL, co-culture experiments were performed in which 5×10^4^ of WT-ADSCs or TRAIL-ADSCs were co-cultured with 5×10^4^ of cell lines CAOV-4, Ej-138, MCF-7, and B16F10. After 48h, the cells were stained with PI. To assay apoptotic potential of TRAIL-ADSCs and effect of IFNγ on sensitivity of melanoma B16F10 cells to TRAIL, 5×10^4^ of either RFP-melanoma or EGFP-melanoma cells were co-cultured with 5×10^4^ of either wild type ADSCs (WT-ADSCs) or TRAIL-ADSCs in 12 well plates. The following day, CM from either GFP-ADSCs or IFNγ-ADSCs was added to the co-cultures and left for 12h and 48h. RFP-melanoma and GFP-melanoma cells were stained with annexin V and PI respectively. BD Cell Quest Pro (BD Biosciences*,* San Jose, CA) and FlowJo (Treestar, Ashland, OR) software’s were used for analysis of FACS data.

**Histological Analyses**

Subcutaneous melanoma tumors and melanoma lung metastases were harvested and 5-μm thick sections of frozen tissues or paraffin sections of formalin fixed tissues were prepared on slides. Tissues were incubated with chicken polyclonal Ab to EGFP (Abcam, Cambridge, MA), rabbit monoclonal anti-mouse IFNγ Ab (Abcam, Cambridge, MA), rabbit polyclonal anti-mouse/human ki67 Ab (DAKO; Hamburg, GDR), rabbit polyclonal anti-mouse PD-L1 Ab (Abcam, Cambridge, MA), rabbit monoclonal anti-mouse CD8 Ab (Serotec Ltd., Oxford, UK), rabbit monoclonal anti-mouse CD4 Ab (Serotec Ltd., Oxford, UK), mouse monoclonal anti-human/mouse FOXP3 Ab (Santa Cruz Biotechnology, Dallas, TX), rat monoclonal anti-mouse CD31 (eBioscience, San Diego, CA) and rat monoclonal anti-mouse IL2 Ab (eBioscience, San Diego, CA) at 1:500 dilution. Following binding with primary antibodies, tissues on slides were incubated with respective horseradish peroxidase-conjugated secondary Abs (Abcam, Cambridge, MA; Leica Microsystems, Newcastle) at 1:1000 dilution. Samples were then incubated with substrate/chromogen, 3,3' – diaminobenzidine (DAB) from Novocastra DAB chromogen and NovoLink DAB Substrate Buffer (Leica Microsystems, Newcastle). The substrate reaction was stopped by washing the slides in water. All tissues were minimally counterstained in hematoxylin. All samples were stained with 0.5% Oil red O, Alician blue, and Alizarin red stainings.

**Supplementary Reference**

1. Skurk T, Ecklebe S, Hauner H. A novel technique to propagate primary human preadipocytes without loss of differentiation capacity. *Obesity*. 2007;15(12):2925-31.

**Supplementary Tables**

**Table S1.** Mean percent of melanoma cell viability measured by MTT assay*

| **Groups** | **Percent of viability** |
| --- | --- |
| **Mock CM** | **1.0000** |
| **Purified**  **IFNγ** | **0.5184** |
| **IFNγ+ CM** | **0.4585** |
| **Control+ (Zeocin)** | **0.2763** |

* MTT assay was performed three days after melanoma cell incubation in CM (culure medium) obtained from either GFP-ADSCs (mock) or IFNγ-ADSCs. Zeocin and purified mouse IFNγ were used as a positive and sample control respectively. MTT = 3-(4,5-Dimethylthiazol-2-yl)-2,5-diphenyltetrazolium bromide, IFNγ = interferon gamma.

**Table S2.** Mean percent of melanoma cell early apoptosis and death (late apoptosis) after 12h and 24 of incubation*

| **IP** | **Apoptosis** | Mock CM | IFNγ+ CM | **Apoptosis** | Mock CM | IFNγ+ CM |
| --- | --- | --- | --- | --- | --- | --- |
| 12h |  | 11.34 ± .91 | 17.59 ± 1.61 |  | 8.33 ± 1.33 | 5.12 ± 1.11 |
|  | **Early** |  |  | **Late** |  |  |
| 24h |  | 16.88 ± 1.21 | 43.09 ± 3.03 |  | 21.60 ± 2.19 | 19.00 ± 2.46 |

* Melanoma cells were incubated with supernatants from either EGFP-ADSCs or IFNγ-ADSCs for 12h and 24h and then they were stained with Annexin-V-FLUOS. IP = incubation period, ADSC = adipose derived mesenchymal stem cell, CM = culture medium; IFNγ = interferon gamma.

**Table S3.** Effect of treatment on the survival of mice with melanoma lung metastases*

| **Survival** | **Groups** | | | | |
| --- | --- | --- | --- | --- | --- |
| Days | Control | EGFP-ADSC | TRAIL-ADSC | IFNγ-ADSC | IFNγ/TRAIL-ADSC |
| Median (95% CI) | 36 | 34 | 33 | 68 | 66 |
| Min to Max | 29 to 39 | 28 to 43 | 25 to 48 | 52 to 86 | 27 to 75 |
| *P* | referent | 0.516 | 0.938 | <0.001 | 0.013 |
| *P* | <0 .001 | < 0.001 | < 0.001 | referent | 0.257 |

* The log-rank test was used to assess the statistical signiﬁcance of differences in survival among the five treatment groups. Pairwise log-rank tests were performed to assess differences in survival among treatment groups of IFNγ-ADSC and IFNγ/TRAIL-ADSC. CI: conﬁdence interval, ADSC = adipose derived mesenchymal stem cell, EGFP = expressed green fluorescent protein, TRAIL = tumor necrosis factor (TNF)-related apoptosis-inducing ligand, IFNγ = interferon gamma.
