# Supplementary figures and images for: PhiC31/PiggyBac modified stromal stem cells: effect of interferon γ and/or tumor necrosis factor (TNF)-related apoptosis-inducing ligand (TRAIL) on murine melanoma
Source: Mol Cancer. 2014 Nov 26;13:255. doi: 10.1186/1476-4598-13-255 (PMC4258801; doi:10.1186/1476-4598-13-255)

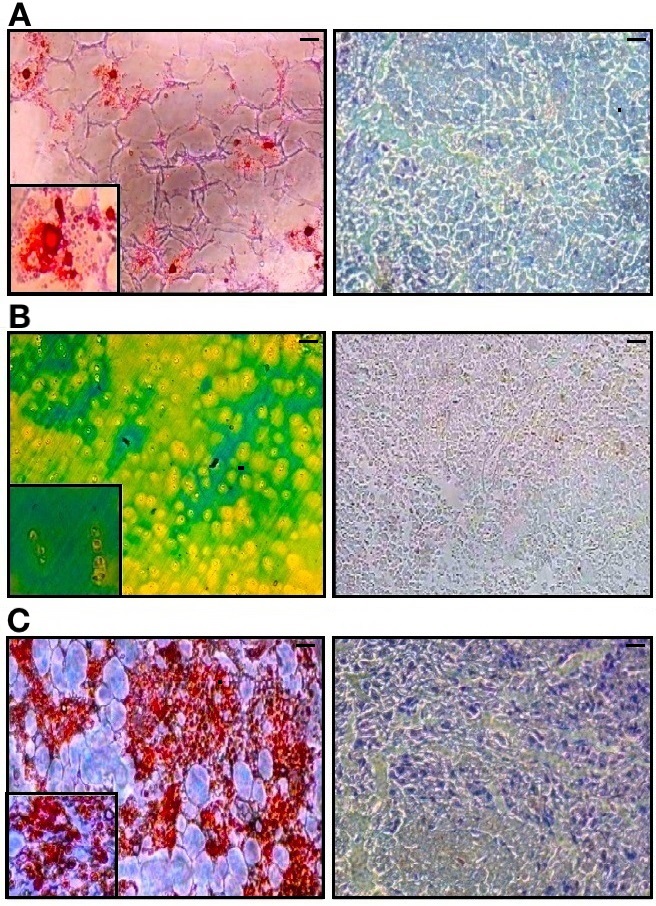

Supplement: Supplementary file 2 — Additional file 2: Figure S1: Differentiation analysis of melanoma tissues. Mouse fat tissue from the inguinal fat pad, murine cartilage isolated from patellae and mouse bone tissue obtained from a 8 week-old male C57BL6 mouse were used as positive controls (Left panels). Representative images from melanoma/IFNγ-ADSCs co-injected groups are shown (Right panels). Results indicated there was no in vivo differentiation of ADSCs from different groups into three mesodermal lineages. Scale bars = 50 μm. A) Oil red O staining. B) Alician blue staining. C) Alizarin red staining. ADSC = adipose derived mesenchymal stem cell, IFNγ = interferon gamma. (JPEG 324 KB) [file 12943_2014_1455_MOESM2_ESM.jpeg]

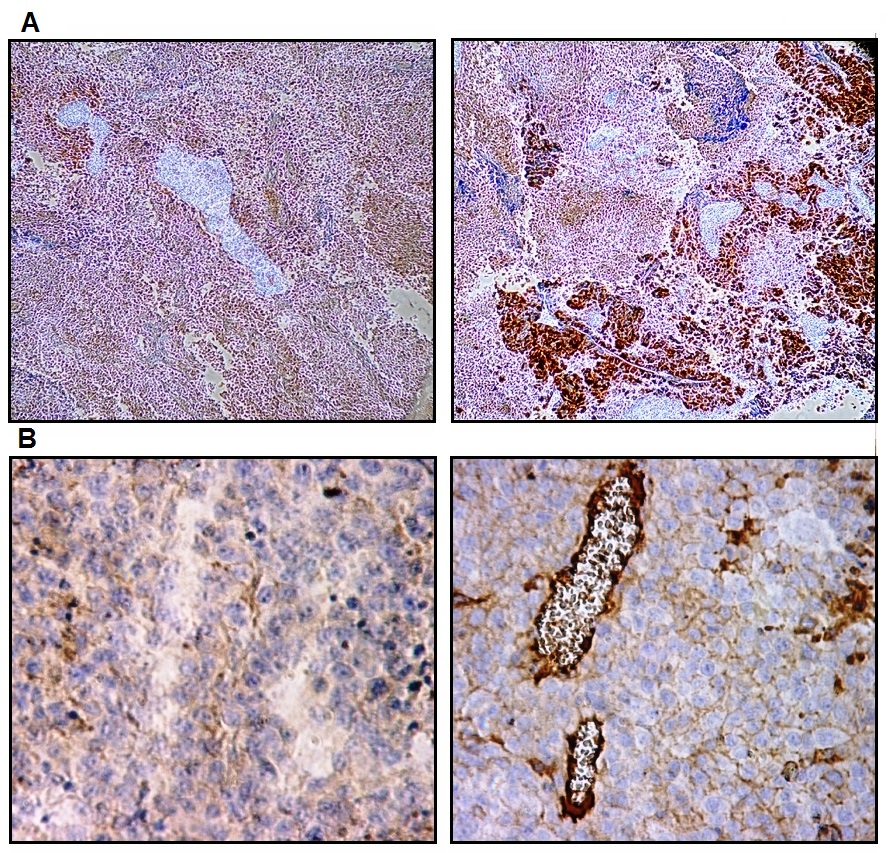

Supplement: Supplementary file 3 — Additional file 3: Figure S2: Formation of capillary-like structures by ADSCs in ADSCs/Melanoma co-injected groups. A) Representative microscopic images of immunohistochemistry (IHC) staining for EGFP. (Left) Melanoma/PBS (Right) Melanoma/EGFP. Capillary-like structures are formed by EGFP expressing ADSCs. B) Representative microscopic images of IHC staining for PD-L1. (Left) Melanoma/PBS (Right) Melanoma/EGFP. Mesenchymal stem cells highly expressing PD-L1 have organized capillary-like structures. (JPEG 523 KB) [file 12943_2014_1455_MOESM3_ESM.jpeg]
